# Local value-chains dedicated to sustainable production (coffee agroforestry business-driven clusters or CaFC): a new organizational model to foster social and environmental innovations through farm renovation

**DOI:** 10.12688/openreseurope.14570.1

**Published:** 2022-05-18

**Authors:** Andrew Meter, Eric Penot, Philippe Vaast, Hervé Etienne, Eric Ponçon, Benoit Bertrand

**Affiliations:** 1 Alliance of Bioversity International and CIAT, Rome, 00153, Italy; 2 UMR Innovation, CIRAD, Université de Montpellier, Montpellier, 34398, France; 3 UMR Eco & Sols, CIRAD, Université de Montpellier, Montpellier, France; 4 UMR DIADE, IRD, CIRAD, Université de Montpellier, Montpellier, France; 5 CIRAD, UMR DIADE, F-34398 Montpellier, France; 6 ECOMTRADING, San José, Costa Rica

**Keywords:** Arabica coffee, agroforestry cluster approach, Nicaragua, Cameroon, Vietnam

## Abstract

**Background: **Worldwide coffee production, especially Arabica coffee, is threatened by climatic change, plants diseases and vulnerability of smallholders. Meanwhile, consumers’ demand for socially and environmentally sustainable products is steadily increasing, driving the engagement of stakeholders in agro-ecological and social initiatives. Here we present a new organizational model, the “Coffee agroforestry business-driven cluster” (CaFC), which aims at preserving ecosystems while offering producers a fair income. Based on an original local micro value-chain dedicated to sustainable production of high-quality Arabica coffee under agroforestry systems, the CaFC model stands out by addressing the issues around plantation renovation, a crucial process that requires considerable investments from producers.

**Methods: **Based on a pilot project in Nicaragua, we illustrate how the operational principles of CaFC can be applied in a real setting. Using data shared by key stakeholders involved in the project, we assess the profitability of the CaFC model by comparing different scenarios and applying sensitivity analysis. We then reflect on the reproducibility of the model in other contexts, building on lessons learned from ongoing implementations in Vietnam and Cameroon.

**Results:** For producers renovating their plantations, the CaFC model consistently outperforms other scenarios, offering high quality premiums coupled with capacity building, access to highly productive varieties that perform well under agroforestry systems and adapted credit with favourable repayment schemes. Implementation in Vietnam and Cameroon show that the model can be successfully replicated with some adaptation to local contexts. These cases also highlight the importance of mutual interests, trust and communication in enabling collaboration between stakeholders.

**Conclusions: **The CaFC model has great potential for positive environmental and economic impact and offers strong incentives for stakeholders involved in its resulting micro value-chain. The concept was initially developed in Nicaragua for coffee but could also be adapted in other countries or even to other commodities such as cocoa.

## Introduction

Arabica coffee ( *Coffea arabica* L.) has established itself as a high-quality product, characterized by its superior organoleptic qualities and considered a social product in many countries where so-called ‘specialty’ coffees have flourished over the last ten years. Producing such coffees involves new specifications for all participants in the supply chain. As the market for specialty coffees is expanding, new issues such as climate change, low carbon food print ( Hergoualc’h *et al*., 2012; Nab & Maslin, 2020) and respect for the environment are emerging.

The objective is now to produce, within the framework of sustainable agriculture, high quality coffee with high yields to guarantee farm profitability, adapted to local climates and respectful of the environment. To preserve biodiversity and limit the effects of climate change, coffee production systems must provide positive externalities to the environment. The changes in production practices needed to achieve these goals often require replanting coffee farms with adapted and efficient varieties. 

Although it is crucial to ensuring the sustainability of their activity, the plantation renovation process can be a huge financial burden for farmers. The investment required to replant one hectare of coffee is in the order of 3,500 to 7,000 USD/ha, depending on the type of planting material (BREEDCAFS survey, Matrice project, personal communications with stakeholders in Nicaragua). Moreover, bank rates available to smallholders through local credit schemes are generally high – between 15 and 25%. Lacking access to the required capital for investment, many producers and especially smallholders tend to postpone this operation. For most smallholders, renovating plantations with the highest quality planting material remains inaccessible.

When developing agroforestry systems, both the issues of plantation renovation and appropriate choice of coffee variety are often overlooked by the research and development community. We argue that these are essential components for improving productivity, quality and sustainability of coffee production systems.

To address this issue, we propose an innovative mode of organization that involves close collaboration between multiple stakeholders, including a strong commitment from the roaster. Indeed, to secure a supply of quality coffee, it is in the roasters' interest to support producers in renovating their plantations with high quality coffee varieties and to therefore remunerate them fairly for implementing agronomic practices that are compatible with the values that they wish to communicate to their customers.

The CaFC (coffee agroforestry business-driven clusters) concept was developed to meet this objective ( Bertrand *et al*., 2019), initially within the framework of a project in 2017 (MATRICE project led by CIRAD), then within the framework of the EU/BREEDCAFS H2020 project initiated in 2019. The aim is to establish agroforestry clusters according to specifications jointly defined by all partners and local actors.

In this article, we first describe the general concept of the CaFC model, its vision and objectives. Based on the pilot project in Nicaragua, initiated in 2016, we illustrate how the CaFC model can be implemented in real conditions and show evidence of its potential. Using data shared by key stakeholders of the Nicaragua project – ECOM (coffee-buyer), the Moringa partnerships fund, NicaFrance (foundation), CIRAD (research institution) and Nespresso (roaster) – as well as data from a similar project in Peru, we assess the profitability of the CaFC model by comparing realistic scenarios and applying sensitivity analysis. We then discuss the model’s reproducibility in other socio-economic contexts, building on lessons learned from the pilot project in Nicaragua and ongoing applications of the model in Vietnam and Cameroon.

### The concept of coffee agroforestry Business driven Clusters (CaFC)

***History and main components of a CaFC*.** Coffee agroforestry Business driven Clusters (CaFC) are defined as local micro value-chains dedicated to sustainable production under agroforestry systems of high-quality Arabica coffee with locally adapted and improved coffee planting material. The principles of these clusters are based on three pillars:

1) The creation of a specific micro-value chain, involving a limited set of actors, makes it possible to maximize gross margin per ha for the benefit of all stakeholders, including farmers. This short and simplified value chain also offers better coffee traceability, an important selling point for the “specialty coffee” market.

2) The use of high-performance coffee varieties that guarantee high yields under agroforestry production systems, resistance to local diseases and high organoleptic quality.

3) Agroforestry management that stabilizes production (over a longer period than full sun plantations), improves and homogenizes coffee quality and provides valuable ecosystem services – through limited use of inputs, increased soil protection, and buffering effects on climate change. It also allows producers to diversify their income by combining the cultivation of coffee with fruit trees or timber trees, depending on local demand and markets.

***Innovation platform and creation of a micro value-chain*.** The CaFC model is based on an original organization orchestrated by a network of six types of stakeholders forming an Innovation and Dialogue platform: producers, roasters, brokers, investors, government organisations and research-development actors. Through this Innovation and Dialogue platform, specifications are agreed upon between stakeholders regarding the varieties of coffee planted, agricultural management, coffee processing as well as purchase prices throughout the chain. Research and development organizations act as third parties in the coordination of the innovation platform, where the objective is to develop an equitable distribution of added value throughout the value chain.

One key point of the CaFC model is that a purchasing price, significantly above that of world market price, is initially accepted by the roaster, guaranteeing all actors downstream to share a significant quality price premium – including participating producers. This reduces risks and allows for the various investments required to develop a system that produces a consistent supply of high-quality coffee. Such a system includes i) renovating plantations with high-quality and high performing locally adapted Arabica plant material, ii) developing agroforestry systems, iii) providing extension services through various means, such as private service providers or digital agriculture information services, to ensure that farmers have the capacity to implement quality-enhancing and agroforestry management practices and adapted processing equipment and services. Eventually for roasters, this commitment enables them to secure a stable supply of quality coffee that is highly differentiated with a high added value potential.

***Plantation renovation at the heart of the concept*.** In order to avoid a drastic decline in productivity, a coffee plantation must be replanted after 15 to 30 years of production – depending on the variety. While this renovation process is crucial to ensuring the sustainability of coffee production, it can be a huge financial burden for farmers.

The cost of replanting itself lies in the order of 3500 to 7000 USD/ha for the first two years according to a 2020 survey implemented by CIRAD in Nicaragua. The majority of coffee is currently produced by smallholders managing less than five ha of coffee ( Jha *et al*., 2011). For these smallholders, generating the capital required to invest in plantation renovation can prove difficult, and local credit mechanisms typically available are not adapted due to excessive interest rates.

Decisions taken during plantation renovation will impact socio-economic and environmental outcomes for years to come. For producers, this process is not only paramount in ensuring the long-term profitability of their farms, it also represents a tremendous opportunity to redefine their production practices, adopt new technologies, and switch to higher quality coffee varieties. Recognizing plantation renovation as a steppingstone in the implementation of sustainable practices, the CaFC model stands out by integrating this process within a tailored and locally adapted sustainable production system, a starting point for which is agroforestry.

***Adopting high quality varieties adapted to agroforestry systems*.** Buyers and roasters are well aware that the choice of variety has a great importance on the sensory profile of the coffee and, ultimately, on its value and marketability. However, with very few exceptions, stakeholders upstream have limited control over the varieties they buy. Moreover, producers often have access to a limited diversity of coffee varieties. The CaFC concept offers a solution to this dilemma: roasters and farmers are given the opportunity to jointly choose a variety and a process tailored to the local production context and with specific characteristics. The decision taken on such an important economic and strategic factor in coffee production, and recognition of common interests between stakeholders is what defines the CaFC model as 'business driven'.

In Arabica, the types of varieties are pure line, clonally propagated F1 hybrid cultivars and F1 hybrid cultivars reproduced by seed (using male gene sterility).

The choice of genetic material is based on three considerations: i) the different agronomic performances between line and hybrid varieties, ii) the possibility of controlling large-scale production of hybrid clones, and iii) the availability of large amounts of seeds.

**The pure lines varieties** are in theory formed by a single homozygous genotype which, through self-fertilisation, gives descendants that are all homozygous and identical to each other and to those of the previous generation.

This type of variety has two advantages: 

a) homogeneity with a maximum performance in a given environment.

b) they are theoretically reproducible in seed form by the user.

The major risk in using line varieties is that of uniformity. Today, 90% of the world's Arabica plantations (more than seven million hectares) are planted with line varieties derived from a narrow genetic basis. Despite an effort of gene introgression, Setotaw *et al*. (2010) showed that the genetic bases of 121 cultivars released in Brazil between 1939 and 2009 were deﬁned by only 13 ancestors.

In the majority of Arabica producing countries, there are no controlled or certified seed production bodies, putting farmers at risk that the seeds for new trees are not of consistent quality, genetic purity and variety standards. Moreover, there is no strict autogamy with Arabica and a certain rate of cross-fertilisation leads to a 'degenerative effect' of the variety after a few generations. With an allogamy rate of 4% to 10%, the percentage of off-types is estimated to be between 12 and 20% after three generations.

The lack of certified seed and seedling producing schemes results sometimes in poor-quality plants or seeds and fraudulent seed sales, which lowers productivity at the farm level. It is therefore necessary to guarantee the quality of planting material with reliable traceability and introduce quality control.

**F1 hybrid cultivars**

F1 hybrid varieties result from the controlled crossing of two distinct homozygous parents.

The F1 hybrid generation obtained between two different gene pools – e.g. American cultivated varieties and Ethiopian wild accessions – yield 30 to 40% more than the best parent ( Bertrand *et al*., 2019).

In Arabica, only the best individual of the best F1 hybrid progenies is selected and cloned by vegetative propagation so as to keep its agronomic performance ( Etienne *et al*., 2018).

The main disadvantage of the vegetative propagation is its elevated price. A cloned hybrid plant, produced in nursery and ready to be field-planted, costs about three times the price of a plant produced by the producer with pure line seeds. Seed production in Arabica F1 hybrids is still a major deterrent; however, the situation becomes less constraining if the hybrid is also produced as seed. 

The technique for F1 hybrid seed production based on seed garden using sterile male individuals was applied on a large scale to produce the first commercial seeds of the F1 hybrid Starmaya ( Georget *et al*., 2019). A Starmaya plant coming out of the nursery and ready to be planted costs about 1.2 times the cost of a plant produced by the producer with pure line seeds. Starmaya F1 hybrids were shown to have better agronomic characteristics in terms of vigor, bean size, and yield than the Marsellesa ^®^ cultivar or the Caturra red as control ( Georget *et al*., 2019; Marie *et al*., 2020). The Starmaya F1 hybrids also had a higher cup quality than the traditional cultivars, such as Marsellesa ^®^ and Caturra red.

Through the CaFC model, high performing and locally adapted F1 hybrids such as Starmaya are developed based on specifications agreed upon between stakeholders of the Innovation platform. Moreover, the quality of the planting material provided through the CaFC cluster is guaranteed through a certification system developed by World Coffee Research – a partner organization in the initiative.

***Offering a fair price*.** Voluntary sustainability standards (VSS) such as Fair Trade, which are becoming a *sine qua non* for access to specialty coffee markets, face increasing criticism from academics and stakeholders of the coffee sector. VSS have been the subject of many studies in the past decades, especially in coffee value chains, and contradicting results have been reported regarding their impacts on various elements of sustainable livelihoods. Whereas some studies show positive effects for at least some environmental, social or economic sustainability indicators, many find no or even negative impacts ( Akoyi & Maertens, 2017; Baake *et al*., 2018; Fort & Ruben, 2017; Minten *et al*., 2018; Mithöfer *et al*., 2017; Oya *et al*., 2017; Vanderhaegen *et al*., 2018). Specialty coffees are usually certified by one or several of these VSS as a communication tool to consumers regarding their sustainability. While they often result in higher prices at the farm gate, questions remain about the extent to which the benefits of higher retail prices translate into higher revenues for farmers and rural communities ( Rueda & Lambin, 2013). As a novel organizational model, CaFC strives to overcome these short-comings. Through transparent collaboration between producers, roasters and other stakeholders, the CaFC model is designed to guarantee shared added value of high quality coffee and fair prices to producers regardless of the size of their farm and volume of coffee delivered.

***Promoting agroforestry systems for increased environmental sustainability*.** The CaFC model addresses one of the most significant threats to coffee production: the shift from diversified shade coffee to simplified shade or unshaded coffee ( Goodall *et al*. 2015; Jha *et al.* 2014).

Coffee agroforests may vary from a single shade tree species planted within the rows of coffee trees to traditional or rustic systems where coffee is planted under managed forests, where numerous tree species provide an almost complete shade cover through a multi-strata tree canopy ( Perfecto *et al*., 2005). A modern version of the latter system is promoted through the CaFC model, where a variety of shade trees are pro-actively selected to provide a range of services to coffee trees. Such systems have been appraised for their services as refuge for biodiversity, including their role in biological corridors, where the productive system also serves to connect fragmented forested areas. Management of shade tree cover in coffee agroforests is also considered one of the key measures towards climate change adaptation ( Vaast *et al*., 2016; Verburg *et al*., 2019). Shade trees in coffee agroforestry systems may serve two climate related purposes: i) adaptation, by buffering the expectedly increasing variability in temperature and rainfall and improving resilience to extreme weather events such as windstorms, frost or hail, and ii) mitigation, by capturing and storing carbon in the shade trees, below and above ground ( Goodall *et al*., 2015; Pinoargote *et al*., 2017; Vaast *et al*., 2016; Verburg *et al*., 2019). Coffee agroforestry systems that have limited disturbance to the soil present a more organic matter-rich system than many other agricultural systems and – when coffee does not replace forests – are a step forward in climate change mitigation ( Guillemot *et al*., 2018; Verburg *et al*., 2019).

Agroforestry systems also provide additional services or benefits to the producers. The shade trees, especially when consisting of flowering trees, may provide a habitat for pollinators and thus provide pollination services, not only to the coffee crop but to other crops in the vicinity ( Boreux *et al*., 2013). The shade environment may also enhance biological control of certain pests and diseases, which otherwise are controlled through commercial or homemade inputs, which come at a cost to farmers. These different services associated with shade trees all contribute to the growth and development of the coffee trees, but the shade trees themselves also directly provide products to the farmers’ households in the form of fuel wood, timber and other materials, as well as food products ( Nguyen *et al*., 2020). Agroforest systems developed through the CaFC model are designed to incorporate native timber trees that increase their resilience to climate change and provide environmental services to neighboring communities. At the end of their life span, these timber trees should represent a significant capital for the producer.

However, the main shortcoming of coffee agroforests remains the associated reduction in coffee yield. Decades of breeding strategies and agricultural extension policies focusing on high yields have resulted in non-shaded coffee monocultures being the dominant production system and has undermined research in varieties adapted to shaded environments and able to perform well under agroforestry systems. This issue is at the core of the CaFC cluster, which renovates farm plantations with new improved Arabica varieties that provide high yield under shaded conditions.

Management of innovative agroforestry systems requires extensive knowledge not always accessible to smallholder farmers. The CaFC model integrates capacity building and training of farmers to overcome these challenges by enabling a central coffee farm to offer technical expertise to smaller farms joining the cluster, as well as an access to innovative processing practices and equipment that can help tackle local sustainability issues. 

***Integrating and creating synergies with current certifications*.** CaFC shares similar objectives with other certification systems – e.g. Fairtrade, Rainforest Alliance, UTZ – offering smallholders access to differentiated markets and price premiums, reducing their vulnerability to price volatility, improving their livelihoods and promoting agricultural practices that are respectful of the environment. The organization in agroforestry clusters does not obliterate these certifications. On the contrary, it uses and reinforces them by increasing their reliability through the improved traceability of the CaFC’s micro-value chain.

***The pilot project in Nicaragua*.** The pilot project in Nicaragua is a great opportunity to illustrate how the CaFC model can be implemented in real life. The pilot cluster was formed in 2016 in Nicaragua, where 1,300 ha of coffee trees are currently cultivated under agroforestry management in association with high quality and high value timber trees. Through this pilot project, coffee producers, ECOM (coffee-buyer), the Moringa partnerships fund, CIRAD and Nespresso (roaster), were all brought together within an Innovation and Dialogue platform coordinated by CIRAD.

Nespresso had initially expressed its interest in a stable supply of high-quality coffee produced by the pilot project in the Matagalpa region of Nicaragua. A cluster of large to medium producers (50 to 150 ha) – referred to as *out-growers –* has been developed around a central coffee estate owned by Nicafrance – La Cumplida. Through this central estate, training and assistance are provided by ECOM and Nicafrance to ensure farmers joining the cluster have the ability to adopt required practices and produce the highest quality coffee cherries. La Cumplida also provides other farms with planting material and access to an innovative processing facility. This pilot project relies on a sustainable processing of the coffee cherries through an innovative wet milling station and applying the *honey process* method. This wet milling station – and others to be built in proximity to small out-growers spread around the cluster – uses far less water and ensures a safe management of wastewater, greatly reducing pressure on water resources and environmental pollution associated with the traditional wet milling process ( Dadi *et al*., 2018). While many farmers have their own wet mills, they do not always have easy access to free water, and many do not adopt best practices regarding waste-water management. Hence, the integration of the wet process through a central (and soon multiple) wet milling station translates into positive environmental impacts while also reducing cost and time for farmers.

Nicafrance and ECOM cover the renovation costs and production costs up to five years after replanting. After five years, large to medium out growers regain full control over their production and reap all the benefits from their new high yielding plantation selling coffee at high prices on a tailored specialty market. They can then pay back the remaining initial costs of renovation (plant material, labour for planting, inputs). Large to medium out-growers are certified UTZ and Rainforest Alliance (these two certification agencies having merged in 2018). 

Since 2017, 24 smallholder farmers – referred to as small out-growers – have joined the cluster with on average one hectare of land renovated with new varieties – mainly the Marsellesa variety. This is a small-scale approach that allows the consortium to cover the risk associated with the experimental use of CaFCs.

Unlike large and medium size coffee farmers, who usually have various income sources and the capacity to recover from various coffee crises (diseases, price volatility, effect of the climate etc.), smallholders solely depend on the income generated by their coffee farm for their livelihood, and the various costs of joining the cluster could be a barrier to entry. A fair contract for smallholders has been developed for the pilot project, which is essential for enabling smallholders to join in the initiative. Smallholders enter into an agreement with ECOM and Nicafrance to lend the land they want to renovate at no cost during the first five years through a usufruct contract. Smallholder’s entry into the initiative is facilitated by tailored credit repayment schemes with a delay in payment the first couple of years. Production costs are repaid by 50% of the coffee production during the years three to five at zero-interest for small out-growers. Renovation costs do not have to be repaid by the farmers during the first five years. In addition, ECOM offers small out-growers access to credit with favourable terms of repayment for them to cover these remaining costs.

The pre-existing relationships between CIRAD and some local large-scale producers have facilitated the current collaboration in Nicaragua. ECOM and Nicafrance have a long-standing business relationship. La Cumplida estate provides labor opportunities to local farmers in the area and Fundacion Nicafrance has established a positive link with the inhabitants around La Cumplida through a long-term project supporting education from primary school to university. Moreover, a clear interest by ECOM and Nicafrance in a sustainable supply of high-quality coffee has led them to accept the level of risks that allow the initiative to develop in the first place. Trust, mutual interest and long-term collaboration greatly facilitated the implementation of CaFC concept and the Innovation platform.

**Research questions**

The impact of joining a CaFC on producers’ net income plays a crucial role in incentivizing farmers and maximizing overall economic, social and environmental benefits. In order to better understand how and to what extent the specificities of the CaFC model can influence the overall income of a hypothetical smallholder, the next section will present a profitability assessment comparing the CaFC model to alternative scenarios.

Another key question relates to the reproducibility of the model in various contexts. Depending on the country, local production practices, challenges faced by coffee farmers and stakeholders, and broader social, economic and political contexts, the CaFC model may need to be adapted. Based on the Nicaragua pilot project, the BREEDCAFS project ( https://www.breedcafs.eu/) has implemented two 10–15 ha clusters in Vietnam and Cameroon. Reviewing these two case studies, along with the Nicaragua prototype, will allow for the identification of key challenges and shortcomings in each context before concluding on key factors that need to be taken into account when applying the model in various contexts.

## Methods

### Profitability assessment

A key selling point of the CaFC model for producers is its attractive economic value in the context of plantation renovation. The impact of joining the CaFC model on producers’ income is the result of multiple overlapping factors including i) quality premiums from roasters, ii) differences in varieties (F1 hybrid - Starmaya, local line variety), implying different costs of production and yields; and iii) differences in credit repayment schemes (CaFC repayment schemes vs local credit schemes).

To assess the profitability of the CaFC model, we compare scenarios in which a producer would renovate one ha of land, through the CaFC model with the F1 hybrid variety ‘Starmaya’, and alternative scenarios involving local varieties and different farm gate prices.

We focus our comparative analysis on a hypothetical agroforestry system under Fairtrade (FT) and Organic certifications, the most common combination between sustainability standards – including in Nicaragua ( Fort & Ruben, 2017; Valkila, 2009). As they are also considered to be the most impactful standards ( Dietz *et al*., 2018) and to complement each other regarding economic, social and environmental sustainability issues ( Parvathi *et al*., 2017), the choice of FT-organic as a baseline to compare with the CaFC model appears the obvious one. We also add a baseline scenario with no certifications (conventional).

***Data sources*.** To assess the profitability of a hypothetical CaFC model, we use available primary data from the Nicaragua pilot project combined with secondary sources for various scenarios.

Data was obtained from ECOM and Nicafrance regarding the costs related to the processing of coffee within the Nicaragua pilot project, and the final purchase price from Nespresso. This allowed us to calculate a realistic farm-gate price within a CaFC model that takes into account the various post-harvest processing costs for producing high-quality and homogeneous green coffee and the associated quality premium guaranteed by the roaster. Moreover, the tailored contract and repayment schemes that have been developed in collaboration with farmers through the Nicaragua pilot project will be used to model the CaFC credit repayment scheme.

As the future of the CaFC model is 100% smallholders and 100% organic, secondary data from a study in Peru considering plantation renovation with different varieties under organic production are used – see Data availability at the end of this document ( Meter *et al*., 2022a). Data used include renovation costs, costs of production and yields under organic production for two varieties: *F1 hybrid – Starmaya* and pure line *Typica*.

Finally, FT-organic price setting mechanisms and amounts of premiums are based on:

- Fair Trade minimum price (1.4 US$/lbs for washed Arabica coffee)
- Organic Price Premium (0.2 US$/lbs)

***Farm gate prices*.** Based on data shared by stakeholders involved in the Nicaragua pilot project, the free on board (FOB) price for green coffee bought by Nespresso is:


CaFCFOB=Cprice+1.03US$/lbs

Processing and handling costs amount to 0.33 US$/lbs of green coffee, which sets the CaFC farm gate price for producers at:


CaFCFGP=Cprice+0.7US$/lbs

Moreover, as we are under the assumption of organic production, we add an extra organic premium to the CaFC farm gate price, set at 0.2 US$/lbs. Eventually, the price received by smallholders under the CaFC model is:


CaFCFGP=Cprice+0.9US$/lbs

For the FT-organic scenario, the farm gate price used is based on the current FT minimum price (1.4 US$/lbs) and organic premium (set at 0.2 US$/lbs). However, if the c-price is above the FT minimum price of 1.4, then the FT organic farm gate price is set base on the c-price and organic premium ( Fort & Ruben (2017)).

When the c-price is below 1.4 US$/lbs, the FT Organic farm gate price is therefore set at:


FTOrganicFGP=1.6US$/lbs

When the c-price is above 1.4 US$/lbs, the FT Organic farm gate price is set at:


FTOrganicFGP=Cprice+0.2US$/lbs

For the conventional scenario, no premiums or minimum price are considered, with a farm gate price equal to the *Cprice*.

For all scenarios, the Cprice is set at 1.1 US$/lbs (average closing price throughout 2020).

***Credit repayment schemes*.** The tailored contract and repayment schemes developed through the Nicaragua pilot project are used for the CaFC model credit repayment scheme. Table 1 summarizes the key modalities of the CaFC repayment scheme, along with typical local credit terms.

**Table 1.** Credit repayment schemes.

	CaFC model repayment scheme	Local credit repayment scheme
Year 1 and 2	No repayment, no costs borne by farmer	Renovation costs are paid back in 5 years, with 20% annual interest
Year 3 to 5	50% of harvest is used to pay off production costs from year 3 to 5	
Year 5 to 10	Remaining renovation costs are paid back in 5 years, with 10% interest	-

***Planting material*.** Plantations in the Nicaragua project have been renovated with dwarf variety Marsellesa®, which produces good quality coffee and is resistant to coffee rust, but with average productivity ( Marie *et al*., 2020). The objective for the next smallholders’ plantations and for the CaFC model in general is to renovate plantations with higher yielding varieties such as Starmaya F1 hybrids, which produce 30% to 50% more than the Marsellesa variety ( Marie *et al*., 2020). In Vietnam and Cameroon, pilot projects are in development using the F1 hybrid variety ‘Starmaya’.

To assess the profitability of the CaFC model, we will therefore consider one ha replanted with high yielding F1 hybrid variety Starmaya. For alternative scenarios, a lower-cost and lower-yielding local variety will be considered.

***Scenarios*.** Table 2 summarizes key parameters that vary in the three scenarios: farm gate prices, credit repayment schemes and varieties used for plantation renovation.

**Table 2.** Summary of scenarios.

Parameter	Scenario 1	Scenario 2	Scenario 3
Variety	F1 hybrid Starmaya	Local variety	Local variety
Price	2 US$/lbs	1.7 US$/lbs	1.1 US$/lbs (Cprice)
Credit scheme	CaFC	Local	Local
Discount rate (r)	10%	10%	10%

Scenario 1 = F1 Starmaya variety, CaFC price (2 US$/lbs), CaFC credit repayment scheme, discount rate = 10%. Scenario 2 = Local variety, FT-Organic price (1.7 US$/lbs), local credit repayment scheme, discount rate = 10%. Scenario 3 = Local variety, Cprice (1.1 US$/lbs), local credit repayment scheme, discount rate = 10%.

***Profitability indicators*.** Assessing the impact of the CaFC model involves projecting streams of costs and benefits that vary throughout the year as coffee productivity varies worldwide from one harvest to another. Moreover, the CaFC model’s proposed credit repayment scheme implies adjusting costs over time. It is therefore important to assess the time differential value of money by discounting costs and revenues using the concept of net present value through *actualization*. Indicators used for profitability assessment are net present value (NPV) and benefit-cost ratio (B/C).


$$NPV=\sum_{t=0}^{t=n}\frac{\left(B_{t}-C_{t}\right)}{{\left(1+r\right)}^{t}}$$

$$B/C=\frac{\sum_{t=0}^{t=n}\frac{\left(B_{t}\right)}{{\left(1+r\right)}^{t}}}{\sum_{t=0}^{t=n}\frac{\left(C_{t}\right)}{{\left(1+r\right)}^{t}}}$$

Where *B _t_* = benefit per ha in each year

*C _t_* = costs per ha in each year

       n = number of periods/years

       r = discount rate

We set the discount rate at r = 10%.

All scenarios were modelled based on available data, with parameters set for each scenario and profitability indicators computed using RStudio – R version 4.1.0 (RRID:SCR_000432). The source code is available publicly ( Meter *et al*., 2022b).

***Sensitivity analysis*.** A sensitivity analysis was applied by measuring NPV under variations of the following parameters:

- Price
- Interest rate of credit
- Discount rate *r*

### CaFC model’s reproducibility in other contexts

While the basic principles of the CaFC model were successfully implemented through the pilot project in Nicaragua, questions remain regarding its scaling up potential and applicability in other regions with different institutional and technological contexts ( Manning & Von Hagen, 2010). Divergent results across studies assessing the impacts of sustainability governance schemes – mainly VSS – could often be partly attributed to differences in social, economic, political or cultural contexts that affect their performance ( Oya *et al*., 2017). Such differences can translate into potential obstacles – but also opportunities – to the development of a similar CaFC model in other contexts. Moreover, different practices and access to technology, and their impacts on the environment, on producer’s income and other stakeholders, affect the model’s potential for impact. For instance, the value of the CaFC model can vary across areas according to differences in swing potential – the difference between the best and worst ways of producing a commodity ( Mithöfer *et al*., 2017).

Along with the pilot project in Nicaragua, we use the case studies of Cameroon and Vietnam to discuss the reproducibility of the model in different contexts and its scaling up potential, and identify key elements to consider when implementing the CaFC model in other contexts.

The three case studies were discussed between the leaders of each project and documented. The Nicaragua case study is described on page 6 of this article. An overview of the Cameroon and Vietnam cases are presented in the following sections.

Important elements of these cases were organized in a table. This exercise led to the identification of key factors enabling the implementation of a CaFC model, organized in categories and presented in the Results section.

***Overview of case study 2: Cameroon*.** In Cameroon, the CaFC model is being exclusively established with Starmaya hybrids. The size of the cluster will initially be based on a far smaller size due to reduced planting material availability with about 20 hectares for potential production between 30 and 40 tons/year. However, a propagation capacity for Arabica F1 hybrids has been installed in Fumbot in Western Cameroon on an IRAD station. The horticultural mini-cutting technique will be transferred to several nurseries including the womens’ nurseries (Njiyoum Women nursery, cooperative COPLACAM) and will allow the cluster to increase in size in a sustainable way. Current domestic production of Arabica coffee does not exceed 3,000 tons/year in Cameroon and the Arabica value-chain is considered as “desperate” after years of decay.

The model offers an integrated approach (production, fair market, socio-economic aspects) defined by common specifications, mutual respect of procedures and the guarantee of 100% traceability. In Cameroon, there are no large producers, but some mid-sized producers (between 10 and 100 hectares) and a vast majority of smallholders often grouped in cooperatives in connection with mid-sized producers and roasters. Coffee would be paid at the same price to all actors, at a level above the world price justifying the change of varieties and production practices in the present context where smallholders are currently discouraged by the low productivity of their variety (the Java variety) and low prices.

Currently the highest quality coffee beans are sold 40 % above world price to two local roasters selling their product in France and locally. Agroforestry systems are still to be defined by the members of the cluster, respecting good quality coffee production constraints. Coffee farmers have switched to other crops, and in particular to annual crops and fruit trees, at the expense of coffee production. Coffee is only “tolerated” as long as it does not compete too much with crops that provide most farmers’ incomes. Camus (2021) has pointed out two different trajectories depending on farmer age. The younger generation is transitioning towards cash crops and fruit tree systems – in particular avocado and safoutier ( *Dacryodes edulis*) – while the older generation prefers trees that bring less income but require minimal management. In Cameroon, a strong local demand on safoutier and avocado show that these fruit trees are not adapted to coffee agroforestry systems as they provide too much shading ( Manga *et al*., 2013). The ' Shade Tree Advise' tool has been updated for this region of West Cameroon. This tool will help guide farmers' choice of shade trees within the agroforestry cluster.

The Bamiléké area is facing a very strong economic pressure towards enhanced productivity. In 2020, the first actors of the future cluster and Innovation and Dialogue platform were identified after two preliminary identification missions ( Penot *et al*., 2018; Penot *et al*., 2019). Local roasters would be associated to the cluster for a niche market (“Cafés André” and “Brûleries modernes”, located in Douala) for this innovative action. Local mid-sized plantations are the “Koutaba monastery” plantation, the “Frères du Noun” plantation, the Bangoua chief and the Foumbot IRAD station with plantations undertaken in 2020. The cluster, initiated in May–July 2020 with the first plantation renovations, was first mainly comprised of small farmers located in the Bangoua, Batoufam, Djutissa, Fongo and Njiyoum communities, along with the aforementioned mid-sized plantations. For further planting in 2021 (around 10,000 trees), new cluster members will be recruited by IRAD from the Foumban, Bafang, Njuttisa, Dschang localities. The cluster remains quite limited but holds great value in terms of demonstration of what could be expected from a new Arabica cluster for farmers very much inclined to abandon coffee in the current production conditions. The new hybrid would provide a real boost in productivity and quality – particularly in agroforestry conditions to which it is adapted – compared to the local Java traditional variety. Successful implementation of the CaFC model in Cameroon could bring new hope to producers and an alternative to the projected demise of national Arabica production within the next 10 years.

In Cameroon, there is a clear common interest from different stakeholders for the production of high-quality coffee especially in the context of a country that is recognized worldwide as a quality origin. Still, attracting international traders is proving difficult and one question remains: Is there a clear incentive for private actors, especially multinationals, in investing in high quality coffee in Cameroon? While CIRAD partners are already present on the ground, a network with other potential partners has to be built, and with it the trust needed for all stakeholders to join in the initiative.

***Overview of case study 3: Vietnam*.** The Vietnamese situation is fundamentally different from the cases of Nicaragua and Cameroon. In Vietnam, almost all the coffee produced is Robusta (Vietnam being the largest Robusta producer in the world). Specialty coffee is an emerging and marginal market. Still, there is some interest in developing the production of quality Arabica, especially in isolated regions of the north that already produce Arabica and on which the BREEDCAFS project is focusing (Northwest regions of Son La and Dien Bien). The social value-chain context appears to be that of mostly isolated smallholders in a form of social and economic atomization as there are very few coffee cooperatives or any forms of farmers’ structuration concerning coffee selling. However, many farms are connected with factories/buyers through informal networks. The current developing mode of farmers’ structuration is based on the process of certification – mainly 4C and UTZ – engaged and highly promoted by mid-size buyer/roaster companies (Mien Tien and Cat Que) and some small size buyers entering the market. Agroforestry systems are based on local fruit trees with coffee, mainly plum, peach and avocado trees in the vicinity of the road and access to market and timber trees in more remote areas ( Nguyen *et al*., 2020).

A small 30 ha coffee cluster is in the process of being created with the Starmaya hybrid comprising the current core of 12 farmers with demo plots and 50 to 60 associated neighbouring farmers that have yet to confirm their interest in planting the new hybrids. As in Cameroon, CIRAD has transferred to its Vietnamese partners the vegetative propagation techniques developed for coffee hybrids (i.e. somatic embryogenesis in *in vitro* culture laboratory and rooted mini-cutting in nurseries) allowing them to keep the agronomic performances of the selected tree. This transfer was done at two public institutions, AGI (SE and rooted mini-cuttings) and NOMAFSI (rooted mini-cuttings). The rooted mini-cuttings cloning technique was then transferred to different private nurseries in the coffee-growing areas of the northwest, i.e. Detech, a private company in Son La; Phuc Sinh, a private trader and roaster in Son La; Mr Hung, a small roaster in Dien Bien; Minh Tien, a mid-sized trader and roaster in Son La. Together, these nurseries have enabled the production of one hundred thousand plants in 2020, allowing the extension of the existing cluster based on demoplots with an additional 30 hectares. This cluster is also likely to grow according to the desire of farmers and roasters. In Vietnam, small size fruit trees such as plum seem to be locally adapted for agroforestry. Further discussions with local smallholders are necessary to develop technical recommendations for associating the right fruit and timber tree species to local context. In this regard, the farmers’ surveys and recent development of the ‘ Shade Tree Advise’ tool in all the three targeted regions in Vietnam, Cameroon and Nicaragua is of great help.

Discussions between local actors are in progress. The current trend towards certification, either UTZ or 4C, should help structure farmers’ organizations and promote the adoption of new hybrid coffee varieties managed in agroforestry systems and management, particularly processing, to improve quality resulting in higher farm gate prices. Finally, different local companies have clearly expressed their interest to be the final roaster/buyer (as is Nespresso in Nicaragua). These stakeholders constitute so far the main actors of the future cluster and associated Innovation and Dialogue platform.

The development of a CaFC in Vietnam could be highly beneficial not only for local smallholders but also in terms of environmental impact given the sustainability crisis faced by the Vietnamese coffee sector. This is due to the excess use of agro-chemicals and the absence of anti-erosion management on very sloppy lands. Hence, the lack of sustainable Arabica coffee produced in Vietnam and the value of the CaFC model in addressing this could become an opportunity if properly communicated. The question remains whether local roasters in Vietnam can lead such a marketing campaign or would need to collaborate with international roasters who may be better equipped to “launch a new origin”.

In Vietnam, a strong network of stakeholders able and ready to develop an organic CaFC has yet to be developed, which will have to take into account the specific institutional and political context. For instance, international companies cannot buy coffee directly from farmers and local actors have to be integrated into the buying scheme. The absence of both large producers and smallholder organizations, the political and regulatory context hindering collaboration with international stakeholders and the full control of the Arabica value-chain by a few local actors call for a different strategy to develop the right incentives and adapted contracts between participants. In northern Vietnam, two companies may have the resources to develop a cluster model: Cat Que and Mien Tien and some others are quite interested. While mutual interest might be the common feature to implement CaFC, trust remains to be built between local actors who are waiting for the first coffee hybrid productions from 2021 to assess its quality and productivity.

Given the many contextual differences between Nicaragua and Vietnam, the ongoing development of the BREEDCAFS project in northern Vietnam will also be a great opportunity to better assess the applicability of the model across various regions and socio-economical contexts.

## Results

### Profitability assessment

**Figure 1.**
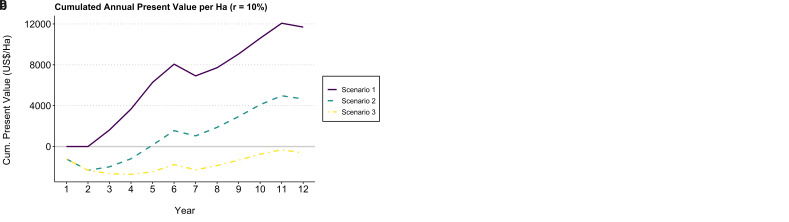
Annual present value ( **a**) and cumulated annual present value ( **b**) per hectare for each scenario from year one to 12. Scenario 1 = F1 Starmaya variety, CaFC price (2 US$/lbs), CaFC credit repayment schemes, discount rate = 10%. Scenario 2 = F1 Local variety, FT-Organic (1.6 US$/lbs), local credit repayment schemes, discount rate = 10%. Scenario 3 = Local variety, Cprice (1.1 US$/lbs), local credit repayment schemes, discount rate = 10%.

***Measuring key profitability indicators*.** Figure 1 displays expected income (annual present value per hectare) and accumulated balance (cumulated annual present value) in the different scenarios.

The variations in results between these different scenarios are explained by three overlapping effects: i) Differences in credit repayment schemes (CaFC repayment schemes vs local credit), ii) Differences in varieties (F1 hybrid – Starmaya, local variety), implying different costs of production and yields; and iii) Differences in farm gate prices.

Results show that coffee production under scenarios 1 and 2 are profitable – positive NPVs – while scenario 3 leads to negative NPV (see Table 3). The first scenario corresponding to the CaFC model with Starmaya comes out as a clear winner with a NPV of 11.694 US$ at year 12 and a B/C ratio of 2.60.

**Table 3.** Results of net present value (NPV) per hectare benefit/cost (B/C) ratio at year 12. *B/C:

	NPV (US$)	B/C ratio
Scenario 1	11694	2.60
Scenario 2	4654	1.38
Scenario 3	-635	0.95

***Sensitivity analysis*.** Table 4 summarizes results of the sensitivity analysis for the three scenarios. Results show that scenario 1 remains more profitable in any case considered.

**Table 4.** Results of net present value (NPV) per hectare at year 12.

	Scenario 1	Scenario 2	Scenario 3
**Cprice variation**			
0.8 US$/lbs	9796	4654	-2750
1.1 US$/lbs	11694 *	4654 *	-635 *
1.5 US$/lbs	15490	5711	3596
**Interest rate** ** variation**			
10%	11694 *	5927	638
20%	10880	4654 *	-635 *
30%	10066	3381	-1908
**Discount rate** ** variation**			
5%	15843	7270	94
10%	11694 *	4654 *	-635 *
20%	6909	1807	-1301

* base value for this scenario

**Price sensitivity**

**Figure 2.**
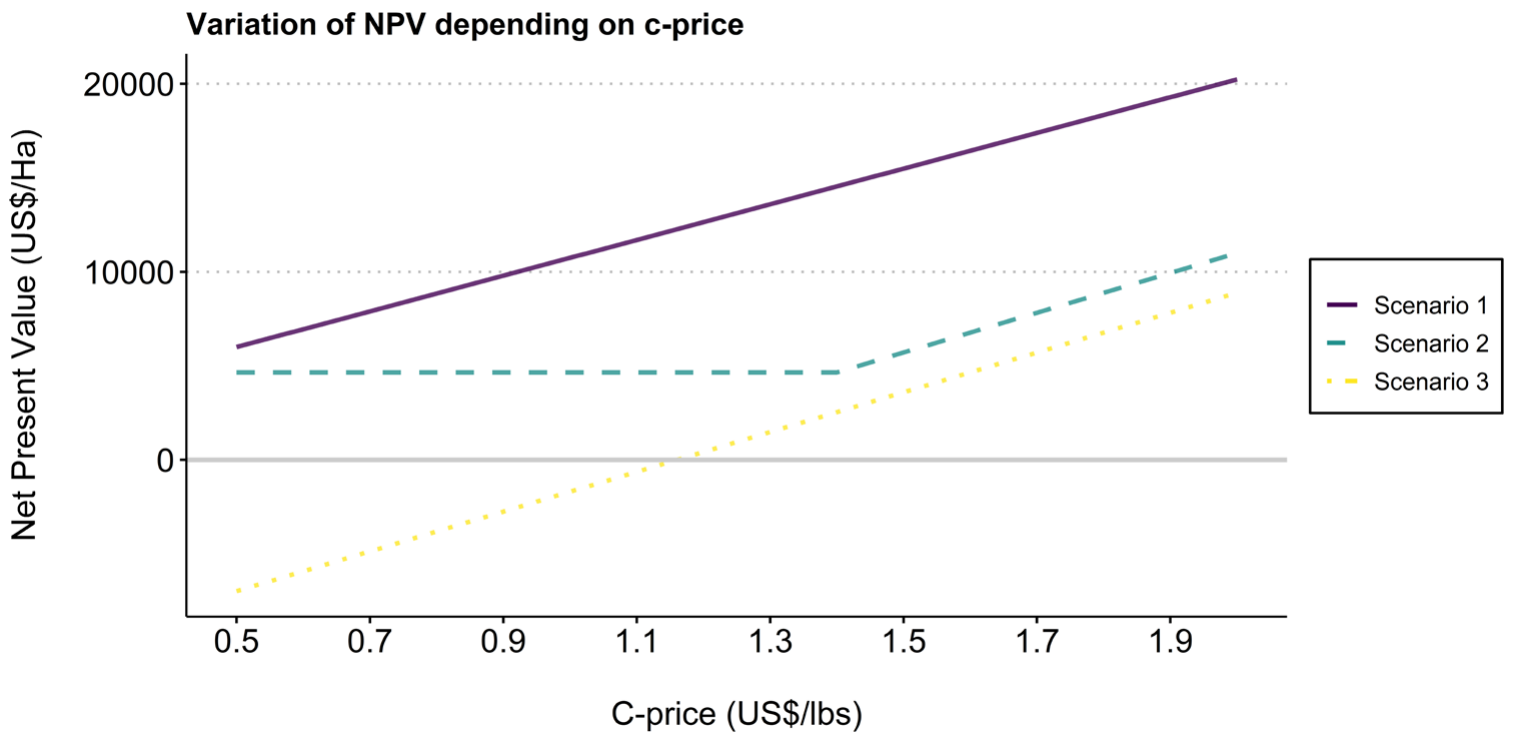
Impact of cprice variation on net present value for each scenario. Scenario 1 = F1 Starmaya variety, CaFC price, CaFC credit repayment schemes, discount rate = 10%. Scenario 2 = Local variety, FT-Organic price, local credit repayment schemes, discount rate = 10%. Scenario 3 = Local variety, bulk price, local credit repayment schemes, discount rate = 10%.

Results of variation in prices show that the FT-Organic scenario outperforms the CaFC model when the Cprice is under 0.5 US$/lbs, a price that has never been reached and is unlikely to be in the near future – see Figure 2.

**Interest rate sensitivity**

In our scenarios, we used an interest rate of 20% for local loans. This appears to be a realistic value in Nicaragua. However, this can vary significantly across countries and regions. It is interesting to note that even with an interest rate lower than that of the CaFC model (<10%), the first scenario still outperforms the two others. Moreover, even at an interest rate of 20%, the NPV for scenario 1 remains over 10,000 US$ per Ha.

**Discount rate sensitivity**

Choosing the right discount rate when calculating NPV is difficult. While we chose to set a discount rate of 10%, this value could change in other contexts where the time-value of money is different. Results from sensitivity analysis show that the relative gap in NPV values between scenario 1 and scenarios 3 and 4 widens as the discount rate increases.

### Reproducibility of the model: lessons learned from the ongoing BREEDCAFS project

The analysis of the three case studies – Nicaragua, Cameroon and Vietnam – led to the identification of several key enabling factors in the implementation of a CaFC model. These factors may have one or multiple dimensions (political, social, economic or technical), and could influence the replicability of the model in multiple ways:

-   Enablement – *complicates or facilitates the implementation of the CaFC model*

-   Adaptation – *requires an adaptation of the model for it to be applicable*

-   Potential for impact – *implies changes in potential for impact of the model, possible tradeoffs*

Table 5 lists the identified key enabling factors for the implementation of the CaFC model in a given area. While not meant to be exhaustive, this list of factors underlines some important challenges and opportunities in the reproducibility of the CaFC model, especially regarding:

**Table 5.** Key factors enabling the implementation of the coffee agroforestry business-driven clusters (CaFC) model in a given target area.

Enabling Factor		Conclusion based on observations in case studies
**National context and policies**		
**Stability of situation**	Is the political and economic situation stable enough to invest in the medium term (5–10 years)?	National political and economic situation may enable or hinder the various interventions needed for implementing CaFC, before and during the implementation.
**Environmental policies**	What are the environmental policies in place in the country?	Positive regulatory and policy environment (e.g. Carbon Credit) can facilitate implementation and increase the value of the model.
**Farmers and collaboration**		
**Farmer typology**	What is the typology of farmers in the target area? What does it imply for the implementation of a CaFC?	CaFC may need to be adapted based on typology of farmers in target area. Potential for positive impact is maximised when CaFC applicable to smallholders.
**Farmer typology**	What is the initial economic situation of farmers in the target area? What does it imply for the implementation of a CaFC?	CaFC may need to be adapted based on economic situation of farmers in target area. Potential for positive impact is maximised when CaFC can significantly improve income.
**Farmers and collective** ** action**	Are farmers in the target area inclined to collaborate? Are cooperatives commonplace?	Reluctance to collective action and low trust between producers in the area will complicate the implementation of the CaFC and might require compensation through other trust-building mechanisms.
**Relationships between** ** producers**	Are there relationship issues or economic inequalities in the target area that could lead to tensions between neighbouring producers?	A negative social context between producers at the local level will complicate the implementation of the CaFC and might require compensation through other trust- building mechanisms.
**CaFC organizational model and stakeholder collaboration**		
**Collective action and ** **relationships between** ** stakeholders**	Are the different actors/stakeholders needed for the development identified? Are they inclined to collaborate?	Initial set of actors needed to implement CaFC Engagement of actors and will to collaborate is important, especially Roaster. Collective action will have impact on results of CaFC throughout.
**Contract acceptability**	Is the CaFC contract acceptable, would it need adaptation?	Depending on political, regulatory and social context, CaFC contract may or may not be acceptable, or may need adaptation.
**Direct contracting**	Is it possible to contract directly with the producer?	Possibility of direct contracting with producers may imply adaptation of contract and relationships with stakeholders.
**Access to credit**	Is agricultural credit available to small producers for replanting?	Depending on credit access, CaFC, its contract and credit terms may need adaptation, and depending on terms available in target area, impact of CaFC may vary.
**Agroforestry systems, diversification, and agronomic practices**		
**Prevalence of ** **agroforestry systems**	Are agroforestry system common farming systems in the target area?	Depending on prevalence of Agroforestry systems in the target area, implementation of CaFC may be easier / need varying levels of technical training. However, if agroforestry systems are not the predominant system in the target area, potential for positive environmental impacts increases.
**Timber trees availability** ** and impact**	Are there local timber trees suitable for agroforestry? May any complication arise from their use?	Availability of local timber trees suitable for agro-forestry will impact design of Agroforestry system, can result in different costs/revenues and environmental impact.
**Fruit trees availability** ** and impact**	Are there fruit trees suitable for agroforestry in the area? May any complication arise from their use?	Availability of fruit trees suitable for agro-forestry will impact design of Agroforestry system, can result in different costs/revenues and environmental impact.
**Pesticide contamination**	Is coffee cultivation accompanied by pesticides contamination?	Depending on current situation in target area regarding pesticide contamination, environmental impact of CaFC will vary.
**Parasitic threats**	Are there specific parasitic threats?	Specific parasitic threats in target area may be incompatible with CaFC, require adaptation of the model, and could influence on long term impacts (e.g. economic through yields, environmental if use of chemicals).
**Productivity**	What are the productivity levels?	Productivity levels are indicative of the leeway for increase in productivity and hence potential for positive impact.
**Extension services**	Do small farmers receive sufficient agronomic support (field visits)?	Quality of extension service would lead to varying levels of technical assistance needed and possible adaptation of the model. Lower quality of existing extension services would translate into potentially high positive impact via the provision of state- of-the-art technical assistance provided to farmers through CaFC.
**Certifications**	What are the main certification schemes in the target area?	Certifications schemes in target area are conducive to adoption of goodproduction practices that can facilitate implementation of CaFC.
**Coffee quality, origin and differentiation strategies**		
**Certifications**	What are the main certification schemes in the target area and their role in differentiation strategy?	Interest in certification schemes as differentiation strategy can be in sync with CaFC.
**Interest in quality** ** improvement**	Is there interest in improving quality among stakeholders?	A clear interest of stakeholders in improving quality of coffee is key for their engagement in the required collaboration for the CaFC model’s implementation.
**Interest in origin** ** differentiation**	Is there interest in promoting the coffee origin of the country of the target area among stakeholders?	An interest in promoting origin differentiation of the target area can further increase mutual interest in quality improvement and the value of the CaFC model.
**Established economic ** **incentives for quality**	Are there economic clear incentives for farmers and stakeholders to improve the quality of their coffee?	Established economic incentives for quality improvement in the target area, such as price differentiation based on quality, can further increase the value of the CaFC model.
**Quality level**	Is the quality level of coffee in the target area satisfactory? Is there room for improvement?	Depending on the general quality level of coffee already produced in the target, the CaFC model could be more or less attractive and imply different levels of impact, with higher impact in areas where general quality is low.
**Breeding strategy and dissemination path**		
**Robusta / Arabica**	What varieties are mainly produced in the target area, Robusta / Arabica?	Production of Robusta vs. Arabica and varieties of Arabica found in the target area will impact the design of the CaFC model and its potential for impact.
**New varieties**	Are stakeholders aware of the value of the new varieties proposed? Is there interest in these new varieties?	Awareness of the quality and value of new varieties proposed through the CaFC would ease implementation. Lack of awareness would imply need to organise demonstration plots etc.
**Propagation capacity**	What is the propagation capacity in the target area? Can the new variety be made easily available?	Propagation capacity impacts scale of implementation. Implies costs for developing propagation capacities (nurseries, training) etc.
**Plantation renovation**	Is there a need expressed by producers in target area for plantation renovation with high quality plant material?	The need from producers for plantation renovation is a pilar of the CaFC model, CaFC model loses its value if implemented in an area where most farms have recently been renovated.
**Plantation renovation**	Are costs of renovation high? Is a solution needed to reduce costs?	Cost of farm renovation in the target area will determine the value of the CaFC model to producers, with higher impacts in areas where renovation costs are high and credit not available to cover them.
**Plantation renovation**	Knowledge on replanting accessible to farmers? Is there a need for technical assistance?	Accessibility to farmers of knowledge on farm renovation in the target area will determine the value of the CaFC model to producers, with higher impacts in areas where technical assistance needed by farmers.

•   National context and policies

•   Farmers and collaboration

•   CaFC organisational model and stakeholder collaboration 

•   Agroforestry systems, diversification, and agronomic practices 

•   Coffee quality, origin and differentiation strategies 

•   Breeding strategy and dissemination pathways

## Discussion

### Environmental and economic value of the CaFC model

The CaFC model has great potential for positive environmental and economic impact. Its strategy based on a homogeneous production of high-quality coffee offers strong incentives for the stakeholders involved in its resulting micro value-chain. Especially for smallholder farmers, high quality premiums coupled with capacity building and access to i) highly productive varieties; ii) renovation of plantations; and iii) adapted credit with favourable repayment schemes make CaFC very attractive. This is reflected by the growing interest of farmers in proximity to the pilot project in Nicaragua that have shared their enthusiasm in joining the next phases of integration and plantation renovations.

Yet, in its current state, the already encouraging pilot project in Nicaragua falls short from an ideal CaFC on some key issues. The aim of CaFC is towards 100% smallholders, 100% organic production and 100% Arabica F1 hybrids used for plantation renovation and management. Results of the profitability assessment of such a hypothetic CaFC model clearly demonstrates its economic value, as the CaFC scenario consistently outperformed the FT-Organic and conventional scenarios. While the results of this exercise are encouraging, further work is needed for a thorough assessment of the economic impact for producers as well as other stakeholders. In this regard, the ongoing extension of the Nicaragua cluster by an extra 350 hectares in an exclusive collaboration with smallholders, under organic certification and using F1 hybrids, is a tremendous opportunity to further assess the benefits of the ideal organic CaFC highlighted in this study.

The environmental value of the model is mostly derived from its application of agroforestry practices, the expected role of which are multiple:

-   Reduction of temperature at the coffee tree level – up to 6°– and create a very buffered growing micro-environment in a context of climatic change ( Vaast *et al*., 2005).

-   Adapt coffee hybrid production potential to a relatively high productivity (comparable to full sun for other local varieties) and a long lifespan or more than 30 years providing therefore short-term and long-term high productivity.

-   Income diversification during coffee production via associated fruit trees or at the end of the lifespan if use of timber trees. 

-   Carbon sequestration by shade trees with a high traceability.

### Reproducibility of the model

The success of the pilot project in Nicaragua is quite context specific. Many factors have come into play to make this project successful. While the CaFC model has great potential for wider application, its reproducibility with comparable success in other contexts may be determined by numerous factors. The comparison of the Nicaragua pilot project and the ongoing application of the model in Vietnam and Cameroon allowed for the identification of key factors enabling the model’s implementation. They include national context and policies; farmer typologies and their socio-economic context; stakeholders, mutual interests in the production of high-quality coffee and potential for collaboration; the status of agroforestry production, local agronomic and technological challenges and specific needs; and breeding strategies, dissemination pathways and challenges in plantation renovation. These factors determine the possibility of replicating the model, enabling or hindering its implementation, but also imply a need for adaptation of the model.

Differences in environmental conditions, climate, local practices etc. requires adapting some elements of the model. For instance, the shade level provided by associate trees has to be adapted to local conditions (generally between 30% and 40%) and take into account local preferences in timber and/or fruit trees. The system is already well defined and well documented in Nicaragua with timber trees, and needs to be adapted with fruit trees. Further discussions with local smallholders are necessary to develop technical recommendations for associating the right fruit and timber tree species to local context. In this regard, the recent development of the ‘ Shade Tree Advise’ tool in all the three targeted countries is of great help.

Different contexts also imply various levels of potential for impacts. In Vietnam, the development of a CaFC cluster could be highly beneficial, not only for local smallholders, but also in terms of environmental impact given the sustainability crisis faced by the Vietnamese coffee sector. In Cameroon, the Arabica coffee value-chain is disappearing if no alternative is provided. CaFC clusters based on new varieties with both increases in price and production could raise a new interest to local smallholders in the Bamiléké area. In this context, the CaFC model stands out as a unique opportunity for local roasters to save Arabica production and the current market, extending its social and economic impact throughout the local value-chain.

An important lesson learned from the cases of Nicaragua, Cameroon and Vietnam is the importance of trust and mutual interest. The success of the on-going cluster in Nicaragua is the result of the collaboration between multiple stakeholders and individuals with mutual interests and relies heavily on trust on at least two levels:

- Trust in the quality of the product and the capacity of various stakeholders to uphold their promises on said quality – e.g. the hybrids developed by CIRAD, the commitment of the farmers to adopt the required agricultural and management practices, the consistency in quality of green coffee resulting from the risky honey-processing.
- Trust in the commitment of all stakeholders in the initiative particularly smallholder farmers who are trusted to hold their end of the agreement regarding land rights and paying back the loan through initial harvests. Producers in turn trust other stakeholders to hold their promises regarding the performance of the hybrids and benefits arising from their adoption as well as their commitment to buy their production at the agreed price.

A clear common interest from different stakeholders to produce high-quality coffee was key in enabling the projects in all three countries, and a pre-requisite for buyers to accept the level of risks that allow the initiative to develop in the first place. Pre-existing relationships with local stakeholders was detrimental in the success of the model in Nicaragua. The presence of CIRAD partners in all three countries was also a strong asset for these initiatives, especially for transferring vegetative propagation techniques developed for coffee hybrids (i.e. somatic embryogenesis in *in vitro* culture laboratory and rooted mini-cutting in nurseries). The case of Nicaragua has also proven the power of demonstration as an important trust-building mechanism, as demo-plots in La Cumplida were critical in convincing smallholder farmers to join the project. Positive results from demo-plots in Cameroon and Vietnam are eagerly awaited by stakeholders to confirm their expectations regarding the productivity of the new varieties to be introduced in the local implementation of the CaFC model.

If the Nicaraguan case proves that mutual interest and trust can bring together large and small producers, a final roaster and a key intermediary organisation (ECOM), it is still questionable whether the CaFC could be applied with different stakeholders, such as with a cooperative of a group of smallholders as a key intermediary organisation. The cluster is very innovative especially regarding the post-harvest processes that allow for the homogeneous production of high-quality coffee. For now, it seems that expertise of some actors (e.g. ECOM) are essential to the success of the initiative and initially to trigger the development of the initiative. However, with insights gained from Nicaragua and ongoing implementation of the CaFC model, perhaps knowledge and technology could be transferred to cooperatives for instance. The answer is probably yes in Nicaragua. In Vietnam, other farmers’ structuration patterns such as certification groups could be used for the same purpose. In Cameroon, the question has yet to be answered.

Eventually, these findings point to the importance of formalizing a Dialog and Innovation platform to ensure transparent communication between stakeholders. In this activity, a methodological proposal has been designed to systematize the BREEDCAFS project in Nicaragua, which is based on the following items: i) provide the history of the project; ii) develop a global reflection and a critical analysis of the successful/not so successful various results of the project, iii) communicate on the experience and iv) collect and use the lessons learned derived from this project to scale up to CaFC including small producers, identifying strategies to adapt this model to the context of small producers based on market demands. The Nicaragua cluster is fully organized and already functional. The objective is to link the current Nicaragua cluster with existing local platforms, associations and other actors in the Arabica sector. Such a Innovation and Dialogue platform has been established as well in both Cameroon and Vietnam for four years where discussions and actions have been engaged with local and international stakeholders.

## Conclusions

The coffee agroforestry business-driven cluster (CaFC) model was developed with the aim of turning the predicament of coffee plantation renovation into a business opportunity by integrating various actors around the plantation and production of coffee with very high organoleptic quality and high added economic value.

The model is based on 1) The creation of a specific micro value-chain, involving a limited set of stakeholders working together to maximize the quality and added value of the coffee produced; 2) The use of high-performance coffee varieties that guarantee high yields under agroforestry production systems, resistance to local diseases and high organoleptic quality; and 3) Agroforestry management that stabilizes production, improves and homogenizes coffee quality and provides valuable ecosystem services.

The CaFC model relies on collaboration, recognition of mutual interests, and trust between the stakeholders involved. In particular, the roaster plays a crucial role by committing itself to buying the product at a high price – between 1.3 and 2 times the standard world price – guaranteeing all actors downstream the share of a significant quality price premium, including participating producers.

The ambition is to establish a sustainable and economically efficient system that could contribute to several global objectives of sustainable development: reduction of social inequalities, fight against global warming, protection of biodiversity, sustainability of agricultural activities.

This paper presented the operational principles of the CaFC model and its potential for positive environmental, social and economic impact. The prototype in Nicaragua helped illustrate the application of the model in a real setting. Especially, results of the model’s profitability assessment clearly shows the economic value of the model for coffee producers that would join a cluster when replanting of coffee trees is needed.

The concept was initially developed in Nicaragua for coffee but could also be developed in other countries or even with other commodities such as cocoa. An analysis of case studies in Nicaragua as well as ongoing implementation in Vietnam and Cameroon allowed for the identification of key enabling factors to consider when implementing the CaFC. A key lesson from the three current cases is the importance of mutual interests and trust between stakeholders: the CaFC model relies heavily on the collaboration between various actors with different motives and objectives, highlighting the importance of using an Innovation and Dialogue platform to ensure communication throughout the process, and trust-building.

While the results of this exercise are encouraging, further work is needed for a thorough assessment of the economic, social and environmental impact of the model as well as its overall value for producers and other stakeholders.

## Data availability

### Underlying data

CIRAD Dataverse: Underlying data for ‘Local value-chains dedicated to sustainable production (coffee agroforestry business-driven clusters or CaFC): a new organizational model to foster social and environmental innovations through farm renovation’, ‘Profitability assessment of the coffee agroforestry business-driven clusters (CaFC)’. https://doi.org/10.18167/DVN1/8RKHFX ( Meter *et al.*, 2022a)

The profitability assessment is based on the following underlying data:

- Data file 1. – BREEDCAFS CaFC: Renovation costs with Starmaya variety.tab
- Data file 2. – BREEDCAFS CaFC: Renovation costs with Local variety.tab
- Data file 3. – BREEDCAFS CaFC: Costs and Yields with Starmaya variety.tab
- Data file 4. – BREEDCAFS CaFC: Costs and Yields with Local variety.tab
- Data file 5. – BREEDCAFS CaFC: Processing costs and buyer price.tab

Data are available under the terms of the Creative Commons Attribution 4.0 International license (CC-BY 4.0).

## Software availability

The R script used for the profitability assessment is available from:

https://github.com/aemeter/CaFC_profitability_assessment.git

Archived source code at time of publication: https://doi.org/10.5281/zenodo.6353652 ( Meter *et al*., 2022b)

License: Common Development and Distribution License 1.0 (CDDL-1.0)
