# Supplementary material for: “« Coffee agroforestry business-driven clusters »: an innovative social and environmental organisational model for coffee farm renovation
Source: Open Res Eur. 2023 Feb 21;2:61. Originally published 2022 May 18. [Version 2] doi: 10.12688/openreseurope.14570.2 (PMC10445845; doi:10.12688/openreseurope.14570.2)
Supplement: Peer review history

# Peer review: Reviewer response for version 2

**DOI:** 10.21956/openreseurope.16963.r30871

**Published:** 2023-03-31

**Authors:** Leonel Lara-Estrada

**Affiliations:** 1 Natural Resources Institute, University of Greenwich, Chatham, UK

The revised version and authors' comments partially address the comments given in the review, but it is enough considering the manuscript topic and circumstances. Thanks.

Is the study design appropriate and does the work have academic merit?

Partly

Is the work clearly and accurately presented and does it cite the current literature?

Partly

If applicable, is the statistical analysis and its interpretation appropriate?

Partly

Are all the source data underlying the results available to ensure full reproducibility?

No source data required

Are the conclusions drawn adequately supported by the results?

Partly

Are sufficient details of methods and analysis provided to allow replication by others?

Partly

Reviewer Expertise:

Coffee production, agroforestry, and trade-off analysis in Central America.

I confirm that I have read this submission and believe that I have an appropriate level of expertise to confirm that it is of an acceptable scientific standard.

# Peer review: Reviewer response for version 2

**DOI:** 10.21956/openreseurope.16963.r30872

**Published:** 2023-02-28

**Authors:** Christophe Montagnon

**Affiliations:** 1 RD2 Vision, Valflaunes, France

The revised version is improved and all comments have been taken into account. Hence, I suggest to Approve for indexing.

Is the study design appropriate and does the work have academic merit?

Yes

Is the work clearly and accurately presented and does it cite the current literature?

Partly

If applicable, is the statistical analysis and its interpretation appropriate?

Yes

Are all the source data underlying the results available to ensure full reproducibility?

Yes

Are the conclusions drawn adequately supported by the results?

Yes

Are sufficient details of methods and analysis provided to allow replication by others?

Yes

Reviewer Expertise:

Coffee agronomy and genetics

I confirm that I have read this submission and believe that I have an appropriate level of expertise to confirm that it is of an acceptable scientific standard.

# Peer review: Reviewer response for version 1

**DOI:** 10.21956/openreseurope.15732.r30060

**Published:** 2022-11-01

**Authors:** Leonel Lara-Estrada

**Affiliations:** 1 Natural Resources Institute, University of Greenwich, Chatham, UK

**General comments**

 The paper introduces a business model (CaFC) to finance the implementation of agroforestry systems using high-performance coffee varieties (high yields & quality and resistance to diseases). The model is a systematization of a past joint experience between public and private entities in Nicaragua. The concept is interesting because it opens the possibility of improving the sustainability of coffee agroforestry systems through a stable and financially attractive scheme for farmers to renovate coffee plantations and sell the coffee produced at a higher price over many years. However, the model's success relies on a particular set of conditions and actors that are at the same time advantages but also constraints for the model replicability and scalability.

 The model relies on using high-performance coffee varieties under agroforestry systems (Principles of the clusters), calling for the multiple benefits of agroforestry, like income diversification, but this is not included in the model evaluation (profitability assessment).

**Specific comments**

**Introduction**

*"The changes in production practices needed to achieve these goals often require replanting coffee farms with adapted and efficient varieties*." What do you mean by replanting coffee farms? Do you mean renovating? If so, this paragraph mentions this is an option "often" chosen by farmers, but in the next paragraph, it mentions that farmers have difficulties to renovate and tend to postpone renovations because of the high investment. Please elaborate.

*"The major risk in using line varieties is that of uniformity*." Please, explain shortly why it is a risk; not all readers may know why.

*"Innovation and Dialogue platform*." It described what topics are discussed and agreed under the platform, but it is unclear what the decision-making "power" of each stakeholder involved is. Do all have the same voice? Please clarify in the text the governance of this platform.

*"This reduces risks and allows for the various investments required to develop a system that produces a consistent supply of high-quality coffee*." A pillar of the model is high coffee quality; studies on the impacts of climate change expect a reduction in the land/climatic suitability for current coffee areas; this was not considered in the profitability assessment; so, I would recommend discussing the possible impacts of climate change on CaFC (yields and quality).

*"…a 'degenerative effect' of the variety after a few generations. With an allogamy rate of 4% to 10%, the percentage of off-types is estimated to be between 12 and 20% after three generations*." Provide a citation/reference.

*"The F1 hybrid generation obtained between two different gene pools – e.g. American cultivated varieties and Ethiopian wild accessions – yield 30 to 40% more than the best parent (Bertrand et al., 2019)."* This reference is for an abstract in a conference, for this statement, a peer reviewed article would be better.

*"Coffee agroforestry systems that have limited disturbance to the soil present a..." *According to Guillemot *et al.* (2018), the change from one coffee agroforestry system to another, changes in tree and coffee plants (varieties), provoke a reduction in the carbon content and, therefore, climate change mitigation potential. CaFC model is about renovating coffee plantations, so the model will cause disturbance above and below the ground during there remotion of old coffee plants and trees and then establish the new coffee plants and shade trees. Discuss the impact of these implications.

 The paper introduces study cases from Nicaragua (1300 ha planted in 2016), Cameroon (20 ha in 2020) and Vietnam (clusters in the process of being created). However, only Nicaragua is a fully implemented case, and the other countries are still in the very early stages (or about to begin) to provide sufficient information on the model performance in those countries.

*"The pilot cluster was formed in 2016 in Nicaragua, where 1,300 ha of coffee trees are currently cultivated..."* Does the 1300 ha were established in 2016, or were they established in previous years? Clarify in the text.

**Methods**

 The profitability assessment evaluates three scenarios where the option using CaFC result the best; from this assessment and scenarios, some questions arise:

1. *Data sources. "To assess the profitability of a hypothetical CaFC model, we use available primary data from the Nicaragua pilot project combined with secondary sources for various scenarios."* Data on postharvest processing costs, coffee price, and financial scheme are from Nicaragua and data on renovating organic coffee plantations from Peru.
  a. Combining data from different countries for this type of analysis might not be the best for consistency: using the costs of one country and coffee prices from another. Discuss why this combination of data, and mention the possible impacts on the profitability assessment.
  b. List the variables used (units and values) from each country.
  c. Are the certification costs included in the analysis (>2year)? They do not appear in the renovation cost files.
  d. Does the data from Peru come from an experiment or plots in different commercial coffee plantations?
  e. The production data (yields) available in the files show that the production values from year 3 to year 6 are the same for the period from year 8 to year 11 (except year 10). Please comment on this. Did the reported data come from actual yields from coffee plantations for the 12 years? If so, the production data are an average of different plantations or came from a single plantation?
  f. Using the same production data provided in the files, I calculated the rate of change (%) from one year to the next for Starmaya and Typica for the 12 years; and it can observe that both varieties have the same rate of change for most years, which is very interesting. I would expect different rates of change. Similar situation for production costs. If I am missing something, please, explain and describe it in the text.
  g. Data from Peru. There is no mention of the biophysical conditions where the coffee plantations were established (altitude, temperature, precipitation, region, etc.); knowing this information would create a clearer picture of the coffee systems under comparison. This information could be added to the text or in Table 2. Summary of scenarios.
  h. All the files in the Data file 1-5 should mention the country of origin in the metadata and the corresponding actual years (e.g. 1996, 1997, etc..) as a column inside each file.
  i. The data on production costs indicate that Starmaya and Local Variety (Typica) has different production costs (≥ 3 years). Starmaya has higher production costs. Why the differences? If comparing both systems, I would assume similar management.
2. It compared the coffee hybrid Starmaya vs the local variety Typica. Why was Typica selected? Its performance is not the best among the traditional varieties or the most used by farmers in the region, such as Caturra. Caturra is widely used in conventional and organic coffee plantations in Nicaragua, where there are 1300 ha under CaFC.
3. Add a short description of Typica in terms of yields, quality, and disease resistance performance; so the reader knows against what is compared to the hybrid Starmaya.
4. *"...with the F1 hybrid variety ‘Starmaya’, and alternative scenarios involving local varieties..."* It is only one local variety.
5. Be specific and use the Typica instead of "Local Variety" in the corresponding figures and tables. There are many local (traditional) varieties with different performances.
6. On the three scenarios that were evaluated, I am not sure they are the best choice because of the narrow comparison, and my impression is that there is not much information to justify the selection. For example, coffee quality is a pillar in CaFC; however, the scenario description does not mention the coffee quality for each scenario; or if the plantations of the three scenarios are supposed to be established at the same location. It is clear that Scenario 1 has high coffee quality and gets the CaFC coffee price premiums (Nesspreso + Organic), but it is unclear what the quality is for Scenarios 2 (FT-Organic) and 3 (conventional). If Scenario 2 and 3 have good coffee quality, they could also participate in Nespresso and get the premiums; otherwise, if the quality of Scenario 2 and 3 is low because they are located under suboptimal conditions, I do not see a point of comparison. This needs to be clarified in the text to avoid a possible misunderstanding of bias in favor of Scenario 1.
7. *Planting material. "Plantations in the Nicaragua project have been renovated with dwarf variety Marsellesa®...(Marie et al., 2020)."* This section is part of the description of the methodology used to do the Profitability assessment, Marsellesa is not part of the scenarios, and the comparison between Marsellesa and Starmaya (already mentioned before in the text) is not ideal because both are relatively new varieties, and Marsellesa is not a local (traditional) variety. This paragraph would be omitted from the text.

**Results**

*"Results from sensitivity analysis show that the relative gap in NPV values between scenario 1 and scenarios 3 and 4 widens as the discount rate increases*." There are only 1, 2, and 3 scenarios.

 Why was the profitability assessment only for 12 years? Why not 30 years, as it is mentioned in the text? Coffee plantations using traditional (local) varieties have a longer lifespan than the one evaluated here; a new renovation is required after 12 years? What is the expected productive lifespan of Starmaya? Even if farmers received favorable conditions to renovate their plantations and received good prices, they expected the plantations to be productive for more than 12 years, and the profitability analysis should consider it.

*"...and a long lifespan or more than 30 years providing therefore short-term and long-term high productivity."* What does it mean for short-term and long-term high productivity?

*"...the hybrids developed by CIRAD..." *for precision, provide the name of the hybrids that this text is referring. Some hybrids were developed in the region by the collaborative effort of many institutions, including CIRAD.

*"Pre-existing relationships with local stakeholders was detrimental in the success of the model in Nicaragua." *Detrimental?

*"...stakeholders. In this activity, a methodological proposal has been designed*." It is not clear what activity you are referring.

**Conclusions**

*"2) The use of high-performance coffee varieties that guarantee high yields under agroforestry production systems..."* on the description of the scenarios include the description of the agroforestry systems (shade tree species, shade level, prunings, etc).

*"3) Agroforestry management that stabilizes production, improves and homogenizes coffee quality..." *Stabilization of the production because agroforestry is not supported in the paper, there is no comparison with full-sun systems to see the such positive effects; and the results on NPV in Figure 1a and the this figure I did using the production data (Data availability section) show that scenario 1 (Starmaya) has similar trend than Typica and a similar rate of change in production from one year to the next (see the image in comments for Methods).

Is the study design appropriate and does the work have academic merit?

Partly

Is the work clearly and accurately presented and does it cite the current literature?

Partly

If applicable, is the statistical analysis and its interpretation appropriate?

Partly

Are all the source data underlying the results available to ensure full reproducibility?

No source data required

Are the conclusions drawn adequately supported by the results?

Partly

Are sufficient details of methods and analysis provided to allow replication by others?

Partly

Reviewer Expertise:

Coffee production, agroforestry, and trade-off analysis in Central America.

I confirm that I have read this submission and believe that I have an appropriate level of expertise to confirm that it is of an acceptable scientific standard, however I have significant reservations, as outlined above.

# Response

**Published:** 2023-02-10

**Authors:** Andrew Meter

**Affiliations:** Alliance of Bioversity International and CIAT, Italy

**General comments**

 The paper introduces a business model (CaFC) to finance the implementation of agroforestry systems using high-performance coffee varieties (high yields & quality and resistance to diseases). The model is a systematization of a past joint experience between public and private entities in Nicaragua. The concept is interesting because it opens the possibility of improving the sustainability of coffee agroforestry systems through a stable and financially attractive scheme for farmers to renovate coffee plantations and sell the coffee produced at a higher price over many years. However, the model's success relies on a particular set of conditions and actors that are at the same time advantages but also constraints for the model replicability and scalability.

 R: We thank the reviewer for his interest in the article and the concept. We agree that the replicability of the model and its scaling is not proven and merits further investigation. We point this out in the perspectives and the last line of the conclusion. Further insight into the replicability of the model will be available soon as a new cluster is currently being set up in Vietnam.

 The model relies on using high-performance coffee varieties under agroforestry systems (Principles of the clusters), calling for the multiple benefits of agroforestry, like income diversification, but this is not included in the model evaluation (profitability assessment).

 R: We agree : In the agroforestry cluster presented here, diversification is done with timber trees. These trees take years to grow and their economic exploitation will have to wait another twenty years.On the other hand, the hidden benefits of agroforestry (preservation of biodiversity, carbon sequestration ..) or the hidden costs of intensification in sunny areas (notably through increased use of pesticides) have not been taken into account because these hidden benefits/costs are very difficult to evaluate in the country context.

**Specific comments**

**Introduction**

*"The changes in production practices needed to achieve these goals often require replanting coffee farms with adapted and efficient varieties*." What do you mean by replanting coffee farms? Do you mean renovating? If so, this paragraph mentions this is an option "often" chosen by farmers, but in the next paragraph, it mentions that farmers have difficulties to renovate and tend to postpone renovations because of the high investment. Please elaborate. R: We agree to reword the sentence as follows : The changes in production practices ideally needed to achieve these goals require renovating coffee farms with adapted and efficient varieties in order to increase the productivity signicantly.

*"The major risk in using line varieties is that of uniformity*." Please, explain shortly why it is a risk; not all readers may know why.

 R : We agree and add : «  The major risk in using line varieties is that of uniformity as they present poor adaptability due to their narrow genetic base and are more prone to the attack of new diseases (Bertrand et al., 2021).»

*"Innovation and Dialogue platform*." It described what topics are discussed and agreed under the platform, but it is unclear what the decision-making "power" of each stakeholder involved is. Do all have the same voice? Please clarify in the text the governance of this platform.

 R : "the dialogue platform also  aims to negotiate the purchase price of coffee. Of course the balance between the stakeholders is delicate and requires a sincere and transparent dialogue."

*"This reduces risks and allows for the various investments required to develop a system that produces a consistent supply of high-quality coffee*." A pillar of the model is high coffee quality; studies on the impacts of climate change expect a reduction in the land/climatic suitability for current coffee areas; this was not considered in the profitability assessment; so, I would recommend discussing the possible impacts of climate change on CaFC (yields and quality).

 R : Indeed, climate change could have negative impacts on the quality of the coffee produced. However, there is no known data on the impact of climate on coffee quality. We therefore prefer not to discuss this impact, which is speculative for the moment.

*"…a 'degenerative effect' of the variety after a few generations. With an allogamy rate of 4% to 10%, the percentage of off-types is estimated to be between 12 and 20% after three generations*." Provide a citation/reference. R : Gallais A. Théorie de la sléection en amélioration des plantes. Coll. Sci. Agron. Masson,Paris. 1989. ISSN : 0336-5247 *"The F1 hybrid generation obtained between two different gene pools – e.g. American cultivated varieties and Ethiopian wild accessions – yield 30 to 40% more than the best parent (Bertrand et al., 2019)."* This reference is for an abstract in a conference, for this statement, a peer reviewed article would be better.

 R : This comment doesn’t apply following changes made to the text based on first reviewer (this part was erased).

*"Coffee agroforestry systems that have limited disturbance to the soil present a..." *According to Guillemot *et al.* (2018), the change from one coffee agroforestry system to another, changes in tree and coffee plants (varieties), provoke a reduction in the carbon content and, therefore, climate change mitigation potential. CaFC model is about renovating coffee plantations, so the model will cause disturbance above and below the ground during there remotion of old coffee plants and trees and then establish the new coffee plants and shade trees. Discuss the impact of these implications.

 R : This remark is interesting, but it leads us into too specific considerations in an already very long article. The paper introduces study cases from Nicaragua (1300 ha planted in 2016), Cameroon (20 ha in 2020) and Vietnam (clusters in the process of being created). However, only Nicaragua is a fully implemented case, and the other countries are still in the very early stages (or about to begin) to provide sufficient information on the model performance in those countries.

*"The pilot cluster was formed in 2016 in Nicaragua, where 1,300 ha of coffee trees are currently cultivated..."* Does the 1300 ha were established in 2016, or were they established in previous years? Clarify in the text.

 R : « The pilot was established intwo years from 2016 to 2017 in Nicaragua.. »

**Methods**

 The profitability assessment evaluates three scenarios where the option using CaFC result the best; from this assessment and scenarios, some questions arise: *Data sources. "To assess the profitability of a hypothetical CaFC model, we use available primary data from the Nicaragua pilot project combined with secondary sources for various scenarios."* Data on postharvest processing costs, coffee price, and financial scheme are from Nicaragua and data on renovating organic coffee plantations from Peru.

 a. Combining data from different countries for this type of analysis might not be the best for consistency: using the costs of one country and coffee prices from another. Discuss why this combination of data, and mention the possible impacts on the profitability assessment.

 R : We agree, however the comparisons between options remains valid. We modified the text accordingly : « To assess the profitability of a hypothetical CaFC model, we use available primary data from the Nicaragua pilot project combined with secondary sources for various scenarios, including production costs data available from cooperative members in Peru. Using production costs from another country is a limitation to our study. However, our objective is to compare the profitability of different replanting scenarios, with an emphasis on the variety and overall organisational model used. Therefore the comparisons remains valid as the cost differentials affect the different scenarios in the same way. »

 b. List the variables used (units and values) from each country.

 R : The list of variables and units used are available in the data files 1 to 5. We have added the country of origin of the data in the title to clarify.

 c. Are the certification costs included in the analysis (>2year)? They do not appear in the renovation cost files.

 R :  No the costs of certification are not included, as they would be the same for all three options.   

 d. Does the data from Peru come from an experiment or plots in different commercial coffee plantations?

 R : The data on production costs from Peru were collected from three cooperatives of more than 1000 farmers each. This information was added in the text.

 e. The production data (yields) available in the files show that the production values from year 3 to year 6 are the same for the period from year 8 to year 11 (except year 10). Please comment on this. Did the reported data come from actual yields from coffee plantations for the 12 years? If so, the production data are an average of different plantations or came from a single plantation?

 R : The production of coffee trees is cyclical and the years 6 and 10 are years of pruning therefore without production. The production data are an average of different plantations.

 f. Using the same production data provided in the files, I calculated the rate of change (%) from one year to the next for Starmaya and Typica for the 12 years; and it can observe that both varieties have the same rate of change for most years, which is very interesting. I would expect different rates of change. Similar situation for production costs. If I am missing something, please, explain and describe it in the text.

 R : These are data observed for the Typica and Starmaya varieties, on several sites in different cooperatives in northern Peru. For Starmaya in addition to greater productivity, a) annual variations are less important, b) production breaks are less important, c) the number of harvests per cycle is higher. However, we favored a 'conservative' comparison based on the Typica cycle.

 g. Data from Peru. There is no mention of the biophysical conditions where the coffee plantations were established (altitude, temperature, precipitation, region, etc.); knowing this information would create a clearer picture of the coffee systems under comparison. This information could be added to the text or in Table 2. Summary of scenarios.

 R : All the data come from the region Norte de Peru’. Region de Jaen and Cajamarca. This information was added in the text.

 h. All the files in the Data file 1-5 should mention the country of origin in the metadata and the corresponding actual years (e.g. 1996, 1997, etc..) as a column inside each file.

 R : The country of origin of the data was added in the title of the files. We do not have the actual years in the original data. i. The data on production costs indicate that Starmaya and Local Variety (Typica) has different production costs (≥ 3 years). Starmaya has higher production costs. Why the differences? If comparing both systems, I would assume similar management.

 R : Since the two varieties have very different production potentials, the input and labor costs are very different.

 It compared the coffee hybrid Starmaya vs the local variety Typica. Why was Typica selected? Its performance is not the best among the traditional varieties or the most used by farmers in the region, such as Caturra. Caturra is widely used in conventional and organic coffee plantations in Nicaragua, where there are 1300 ha under CaFC.

 R : That's a good point by the reviewer. Typica is used here as a type of coffee not very improved which is also a historical reference still often used in many producing countries (Mexico, Peru, Jamaica for the Blue mountain, etc...). Added this in the text.

 Add a short description of Typica in terms of yields, quality, and disease resistance performance; so the reader knows against what is compared to the hybrid Starmaya.

 R : Added this information in the text. 

*"...with the F1 hybrid variety ‘Starmaya’, and alternative scenarios involving local varieties..."* It is only one local variety. R : We change the sentence «  with the F1 hybrid variety ‘Starmaya’, and alternative scenarios involving a local pure line variety..." 

 Be specific and use the Typica instead of "Local Variety" in the corresponding figures and tables. There are many local (traditional) varieties with different performances.

 R : We agree and replaced « Local variety » with « Typica » in our analysis of scenarios 

 On the three scenarios that were evaluated, I am not sure they are the best choice because of the narrow comparison, and my impression is that there is not much information to justify the selection. For example, coffee quality is a pillar in CaFC; however, the scenario description does not mention the coffee quality for each scenario; or if the plantations of the three scenarios are supposed to be established at the same location. It is clear that Scenario 1 has high coffee quality and gets the CaFC coffee price premiums (Nesspreso + Organic), but it is unclear what the quality is for Scenarios 2 (FT-Organic) and 3 (conventional). If Scenario 2 and 3 have good coffee quality, they could also participate in Nespresso and get the premiums; otherwise, if the quality of Scenario 2 and 3 is low because they are located under suboptimal conditions, I do not see a point of comparison. This needs to be clarified in the text to avoid a possible misunderstanding of bias in favor of Scenario 1.

 R : The higher quality of the coffee produced under the CaFC model is the result of the collaboration between stakeholders and various investments put into the variety used in farm renovation, processing equipment and protocols that result in a coffee with a specific flavour profile. The quality premium offered by Nespresso is therefore only available to participating producers. Producing higher quality coffee does not necessarily translate into higher prices without access to higher value markets that will pay for it. Hence, an original assumption in our scenarios 2 and 3 is that producers outside of the CaFC model do not have access to quality premiums and sell their coffee at the international coffee price (c-price). Scenario 2 also includes Fair trade and Organic benefits (FT minimum price and Organic premium). While the quality premium offered by Nespresso is relatively high, the final price within the CaFC scenario is still based on the c-price and subject to its volatility. This is why comparing it to a Fair-trade organic scenario is interesting, as it includes the benefit of the Fait trade minimum price if the c-price goes below a certain threshold – see results of sensitivity analysis.

*Planting material. "Plantations in the Nicaragua project have been renovated with dwarf variety Marsellesa®...(Marie et al., 2020)."* This section is part of the description of the methodology used to do the Profitability assessment, Marsellesa is not part of the scenarios, and the comparison between Marsellesa and Starmaya (already mentioned before in the text) is not ideal because both are relatively new varieties, and Marsellesa is not a local (traditional) variety. This paragraph would be omitted from the text.

 R : In deed Marsellesa is not part of the scenarios. This is mentioned to clarify that the pilot cluster in Nicaragua was initiated using Marsellesa, but is now ongoing using Starmaya variety – as is the case for projects in Vietnam and Cameroon. The use of high performing varieties such as Starmaya is also the objective for future implementations of the CaFC model. Therefore, the Starmaya variety and associated costs/yields is used in the CaFC scenario, and compared to Typica in scenarios 2 and3.

**Results** *"Results from sensitivity analysis show that the relative gap in NPV values between scenario 1 and scenarios 3 and 4 widens as the discount rate increases*." There are only 1, 2, and 3 scenarios.

 R : Corrected to «…  and scenarios 2 and 3 widens…».

 Why was the profitability assessment only for 12 years? Why not 30 years, as it is mentioned in the text? Coffee plantations using traditional (local) varieties have a longer lifespan than the one evaluated here; a new renovation is required after 12 years? What is the expected productive lifespan of Starmaya? Even if farmers received favorable conditions to renovate their plantations and received good prices, they expected the plantations to be productive for more than 12 years, and the profitability analysis should consider it.

*"...and a long lifespan or more than 30 years providing therefore short-term and long-term high productivity."* What does it mean for short-term and long-term high productivity?

 R : We agree that a coffee plantation should be established for more than 12 years. We expect a useful life of more than 15 years at a minimum and even traditionally up to 25-30 years. However, recent coffee rust crises have destroyed many of the traditional plantations of Typica, Bourbon, Caturra or Catuai in Central America. They are replaced today by plantations of Sarchimors (Obata, Paraiso, T5175, Marsellesa, etc.); the lifespan of these plantations is not yet sufficient to estimate their useful life. As for the F1 hybrids, the plantations that were made in Costa Rica from 2003 and in Nicaragua from 2005, show that their useful life is much longer than those of pure lines. In fact what we demonstrate here over 12 years should be even more true after 20 years. However, we considered that 12 years was a sufficient period of time to draw solid conclusions from our study.

*"...the hybrids developed by CIRAD..." *for precision, provide the name of the hybrids that this text is referring. Some hybrids were developed in the region by the collaborative effort of many institutions, including CIRAD

 R : We removed the mention of 'hybrids developed by CIRAD' as replaced by “the performance of the new varieties for plantation renovation”.

*"Pre-existing relationships with local stakeholders was detrimental in the success of the model in Nicaragua." *Detrimental?

 R : In deed not the right choice of word, Modified text as follows « Pre-existing relationships with local stakeholders was a great advantage participating in the success of the model in Nicaragua.” *"...stakeholders. In this activity, a methodological proposal has been designed*." It is not clear what activity you are referring. R30/ Clarified that “this activity” is referring to the development of Dialog and Innovation platforms.

**Conclusions**

*"2) The use of high-performance coffee varieties that guarantee high yields under agroforestry production systems..."* on the description of the scenarios include the description of the agroforestry systems (shade tree species, shade level, prunings, etc).

*"3) Agroforestry management that stabilizes production, improves and homogenizes coffee quality..." *Stabilization of the production because agroforestry is not supported in the paper, there is no comparison with full-sun systems to see the such positive effects; and the results on NPV in Figure 1a and the this figure I did using the production data (Data availability section) show that scenario 1 (Starmaya) has similar trend than Typica and a similar rate of change in production from one year to the next (see the image in comments for Methods).

 R : Added this in the introduction ; “The shade is composed of native species that have proven themselves in agroforestry systems, associated with coffee (Cordia alliodora ‘Laurel’, Swietenia humilis ‘Caoba’, Juglans olanchana ‘Noga’l, Platymiscium pleiostachyum ‘Coyote’), with densities between 250 and 400 trees per hectare at the time of replanting. During the first 5 years of development, thinning leads to real densities of 200 to 350 trees/hectare The percentage of shade at noon on a sunny day varies between 25 and 40% depending on the plots (exposure and age of trees). “

# Peer review: Reviewer response for version 1

**DOI:** 10.21956/openreseurope.15732.r29316

**Published:** 2022-05-25

**Authors:** Christophe Montagnon

**Affiliations:** 1 RD2 Vision, Valflaunes, France

This article presents an innovative business driven local value chain based on a trustful partnership involving different stakeholders: farmers, research, financial entity, trader, roaster. The paper is well written, brings novelty and is based on good framework of analysis and data. The discussion and conclusions are well supported.

*General comments:*

**Title **is too long. It needs to be shortened. I would suggest the word "innovative" be in the title.

**Literature**: Literature review is good in general. However, it would be good to add some recent publications of Dietz and/or Grabbs, such as "Dietz, T., & Grabs, J. (2022). Additionality and implementation gaps in voluntary sustainability standards. *New political economy*, *27*(2), 203-224" ^1^. Indeed, it would naturally support the CaFC.

**Varieties (1)**: CaFC are supposed to be using improved or "high performance coffee varieties", as stated in the conclusion. The paper states that F1 hybrids are the appropriate varieties. While this sounds correct, it is not necessary to equate CaFC to F1 hybrids but rather to "high performance varieties", what ever they are from a breeder's perspective. It is fair to explain in the beginning of the paper why the choice is to use F1 hybrids as the best available performing varieties, but then in the text and discussion / conclusion you might be more generic on using the best performing available varieties. 

**Varieties (2)**: A side effect on referring to F1 hybrids is that the word "hybrid" is used all along the paper, sometimes not consistently (F1 or F1 hybrid or hybrid or even Starmaya hybrid). Apart from inconsistency, the word "Hybrid" might be misinterpreted. What is important is that the variety is resilient, adapted to shade and accessible. The last part is specific to "F1 hybrid". However, the important part is to build a good local seed or cuttings sector. I suggest you search for "hybrid" in your text and make it consistent. My recommendation is to use high performance varieties after you first explain that the highest performance variety in your case if a variety that happens to be a F1 hybrid. You can choose otherwise, but be consistent.

**Research and farming practices in CAFS**: You should insist more that in fact there is not much of literature that gives clear recommendation and Ag practices for CAFS system and even less related to profitability. it does not change your paper and initiative, but you should insist on the need of simple, realistic applied research.

**"Do nothing" scenario**: I was wondering why you did not add a "Do nothing" scenario, that is don't renovate and continue. At least, you should explain your choice.

**Table 5 - **Table 5 should be improved or even rethought. It is very difficult to read and not much informative. You list issues and basically tell that this issue is to be taken into account depending on the context. It would be more important to the reader to give your top issues (like the top 5) and give more explanations on how they can be key factors of success or failure.

*Details:*

 "High quality": You should give your definition of "High quality coffee" and "High quality" planting material. 

*"The changes in production practices needed to achieve these goals often require replanting coffee farms with adapted and efficient varieties". *Give a reference and explain what is an efficient variety (see general comments on variety).

*"2) The use of high-performance coffee varieties that guarantee high yields under agroforestry production systems, resistance to local diseases and high organoleptic quality."  *Resistance to local diseases is not necessarily true for F1 hybrids but their vigor allows to cope with susceptibility (see Cirad references, Toniutti *et al*. ^2^). Maybe you might use the word "resilient" varieties?

 " *In order to avoid a drastic decline in productivity, a coffee plantation must be replanted after 15 to 30 years of production – depending on the variety*". Please be cautious in that statement or support it with literature review. I have not seen robust support to that statement. However, it is very likely that sustainable practices in CaFC would allow to increase the lifespan of a coffee plot.

*"The cost of replanting itself lies in the order of 3500 to 7000 USD/ha for the first two years according to a 2020 survey implemented by CIRAD in Nicaragua." *Are you considering the first two years here because of the time for the first harvest? Which is highly depending on variety. Please be precise.

***"Adopting high quality varieties adapted to agroforestry systems". *** All this section ending just before " ***Offering a fair price", *** should be improved. You seem to oppose fixed lines and F1 hybrids (which is a debate in many dimensions). Why not just tell you need the best available variety and that in your case, given the status of art, the best available variety happen to be modern varieties selected under a F1 hybrid breeding programme? Then of course, you will mention the caveat of F1 hybrids related to their genetic make up. But recent progress / Starmaya and superiority of these varieties is worth it. 

*"Today, 90% of the world's Arabica plantations (more than seven million hectares) are planted with line varieties derived from a narrow genetic basis" *You might cite my paper: Montagnon, C., Mahyoub, A., Solano, W., & Sheibani, F. (2021). Unveiling a unique genetic diversity of cultivated Coffea arabica L. in its main domestication center: Yemen. Genetic Resources and Crop Evolution, 68(6), 2411-2422 ^3^.

*"In the majority of Arabica producing countries, there are no controlled or certified seed production bodies, putting farmers at risk that the seeds for new trees are not of consistent quality, genetic purity and variety standards"* You might cite my paper: Pruvot-Woehl, S., Krishnan, S., Solano, W., Schilling, T., Toniutti, L., Bertrand, B., & Montagnon, C. (2020). Authentication of Coffea arabica varieties through DNA fingerprinting and its significance for the coffee sector. *Journal of AOAC International*, *103*(2), 325-334 ^4^.

*"In Arabica, only the best individual of the best F1 hybrid progenies (...)"* : individual(S)

*"A Starmaya plant coming out of the nursery and ready to be planted costs about 1.2 times the cost of a plant produced by the producer with pure line seeds"*. I think you mean that it is cheap compared to clonal plants. This should be precise.

*"Voluntary sustainability standards (VSS) such as Fair Trade, which are becoming a sine qua non for access to specialty coffee markets, (...)"*: "Sine qua non" is a little bit strong. "are often associated with" sounds more correct.

*"the shift from diversified shade coffee to simplified shade or unshaded coffee ( Goodall et al. 2015; Jha et al. 2014)"*.: I think there are more recent publications such as Harvey, C. A., Pritts, A. A., Zwetsloot, M. J., Jansen, K., Pulleman, M. M., Armbrecht, I., ... & Valencia, V. (2021). Transformation of coffee-growing landscapes across Latin America. A review. *Agronomy for sustainable development*, *41*(5), 1-19 ^5^.

  " *La Cumplida also provides other farms with planting material and access to an innovative processing facility."* The point here is "High quality" planting material. 

*"(...) smallholders are currently discouraged by the low productivity of their variety (the Java variety) and low prices." *Be cautious on the Java variety which is not a poor variety in other context. Farming practices and neglecting the coffee plots is the main issue here.

*Figure 1a.* You might explicit in the text the reason why the NPV is zero for scenario 1 in the first two years. It is one of the main characteristic of this scenario.

 + in the capture you mention "F1 local variety" for scenario 2. 

*Table 4*. Is the title correct?

 (Discussion)

*"Especially for smallholder farmers, high quality premiums coupled with capacity building and access to i) highly productive varieties; ii) renovation of plantations; and iii) adapted credit with favourable (...)*." Be consistent with your qualification of varieties 'Highly productive"? Or "highly performing"? Or "Shade adapted"? Or other (resilient)? I think "Highly productive" is not giving justice to the message of the paper. 

*"Differences in environmental conditions, climate, local practices etc. requires adapting some elements of the model. For instance, the shade level provided by associate trees has to be adapted to local conditions (generally between 30% and 40%) and take into account local preferences in timber and/or fruit trees. " *See my general comment of Ag practices which are not well documented and for which applied research is needed. 

*"Pre-existing relationships with local stakeholders was detrimental in the success of the model in Nicaragua." *Detrimental? 

*"a final roaster and a key intermediary organisation (ECOM),"* In fact, Ecom is the trader. Indeed, trader is at the interface between farmers and roasters.

Is the study design appropriate and does the work have academic merit?

Yes

Is the work clearly and accurately presented and does it cite the current literature?

Partly

If applicable, is the statistical analysis and its interpretation appropriate?

Yes

Are all the source data underlying the results available to ensure full reproducibility?

Yes

Are the conclusions drawn adequately supported by the results?

Yes

Are sufficient details of methods and analysis provided to allow replication by others?

Yes

Reviewer Expertise:

Coffee agronomy and genetics

I confirm that I have read this submission and believe that I have an appropriate level of expertise to confirm that it is of an acceptable scientific standard, however I have significant reservations, as outlined above.

## References

[1] : Additionality and Implementation Gaps in Voluntary Sustainability Standards. *New Political Economy* .2022;27(2) : 10.1080/13563467.2021.1881473 203-224 10.1080/13563467.2021.1881473

[2] : Influence of Environmental Conditions and Genetic Background of Arabica Coffee (C. arabica L) on Leaf Rust (Hemileia vastatrix) Pathogenesis. *Front Plant Sci* .2017;8: 10.3389/fpls.2017.02025 2025 10.3389/fpls.2017.02025 29234340PMC5712408

[3] : Unveiling a unique genetic diversity of cultivated Coffea arabica L. in its main domestication center: Yemen. *Genetic Resources and Crop Evolution* .2021;68(6) : 10.1007/s10722-021-01139-y 2411-2422 10.1007/s10722-021-01139-y

[4] : Authentication of Coffea arabica Varieties through DNA Fingerprinting and its Significance for the Coffee Sector. *J AOAC Int* .2020;103(2) : 10.1093/jaocint/qsz003 325-334 10.1093/jaocint/qsz003 33241280

[5] : Transformation of coffee-growing landscapes across Latin America. A review. *Agron Sustain Dev* .2021;41(5) : 10.1007/s13593-021-00712-0 62 10.1007/s13593-021-00712-0 34484434PMC8406019

# Response

**Published:** 2023-02-10

**Authors:** Andrew Meter

**Affiliations:** Alliance of Bioversity International and CIAT, Italy

*General comments:*

**Title **is too long. It needs to be shortened. I would suggest the word "innovative" be in the title.

 R : We agree and change the title as follow :

 « Coffee agroforestry business-driven clusters »: an innovative social and environmental model organization for coffee farm renovation

**Literature**: Literature review is good in general. However, it would be good to add some recent publications of Dietz and/or Grabbs, such as "Dietz, T., & Grabs, J. (2022). Additionality and implementation gaps in voluntary sustainability standards. *New political economy*, *27*(2), 203-224" ^1^ . Indeed, it would naturally support the CaFC.

 R : We agree and thank the reviewer for this reference that we add.

**Varieties (1)**: CaFC are supposed to be using improved or "high performance coffee varieties", as stated in the conclusion. The paper states that F1 hybrids are the appropriate varieties. While this sounds correct, it is not necessary to equate CaFC to F1 hybrids but rather to "high performance varieties", what ever they are from a breeder's perspective. It is fair to explain in the beginning of the paper why the choice is to use F1 hybrids as the best available performing varieties, but then in the text and discussion / conclusion you might be more generic on using the best performing available varieties.

 R : We agree and have modified the text accordingly

**Varieties (2)**: A side effect on referring to F1 hybrids is that the word "hybrid" is used all along the paper, sometimes not consistently (F1 or F1 hybrid or hybrid or even Starmaya hybrid). Apart from inconsistency, the word "Hybrid" might be misinterpreted. What is important is that the variety is resilient, adapted to shade and accessible. The last part is specific to "F1 hybrid". However, the important part is to build a good local seed or cuttings sector. I suggest you search for "hybrid" in your text and make it consistent. My recommendation is to use high performance varieties after you first explain that the highest performance variety in your case if a variety that happens to be a F1 hybrid. You can choose otherwise, but be consistent.

 R : We agree and have modified the text accordingly

**Research and farming practices in CAFS**: You should insist more that in fact there is not much of literature that gives clear recommendation and Ag practices for CAFS system and even less related to profitability. it does not change your paper and initiative, but you should insist on the need of simple, realistic applied research.

 R : We agree and have modified the text accordingly

**"Do nothing" scenario**: I was wondering why you did not add a "Do nothing" scenario, that is don't renovate and continue. At least, you should explain your choice.

 R : As most farms are de facto insolvent, we believed that the "do nothing" was explicit. We did not consider the 'Do nothing' scenario' because without replanting the farm is considered unprofitable by the stakeholder. We added an explanation in the text

**Table 5 - **Table 5 should be improved or even rethought. It is very difficult to read and not much informative. You list issues and basically tell that this issue is to be taken into account depending on the context. It would be more important to the reader to give your top issues (like the top 5) and give more explanations on how they can be key factors of success or failure.

 R : This table represents a grid for evaluating the feasibility of a cluster. We don't think that sorting according to priorities would bring more readability.

*Details:*

 "High quality": You should give your definition of "High quality coffee" and "High quality" planting material. 

*"The changes in production practices needed to achieve these goals often require replanting coffee farms with adapted and efficient varieties". *Give a reference and explain what is an efficient variety (see general comments on variety).

 R : we modified the text according to the suggestion of the reviewer. We changed ‘high quality ‘ by ‘Specialty coffee’ and « High quality planting material’ by high performance varieties

*"2) The use of high-performance coffee varieties that guarantee high yields under agroforestry production systems, resistance to local diseases and high organoleptic quality." *Resistance to local diseases is not necessarily true for F1 hybrids but their vigor allows to cope with susceptibility (see Cirad references, Toniutti *et al*. ^2^ ). Maybe you might use the word "resilient" varieties

 R : we modified the text according to the suggestion of the reviewer

 " *In order to avoid a drastic decline in productivity, a coffee plantation must be replanted after 15 to 30 years of production – depending on the variety*". Please be cautious in that statement or support it with literature review. I have not seen robust support to that statement. However, it is very likely that sustainable practices in CaFC would allow to increase the lifespan of a coffee plot. R : we modified the text according to the suggestion of the reviewer

*"The cost of replanting itself lies in the order of 3500 to 7000 USD/ha for the first two years according to a 2020 survey implemented by CIRAD in Nicaragua." *Are you considering the first two years here because of the time for the first harvest? Which is highly depending on variety. Please be precise.

 R : This range is based on calculations made taking into account low yielding varieties like Typica or high yielding varieties like the H1 hybrid 'centroamerica'

***"Adopting high quality varieties adapted to agroforestry systems". *** All this section ending just before " ***Offering a fair price", *** should be improved. You seem to oppose fixed lines and F1 hybrids (which is a debate in many dimensions). Why not just tell you need the best available variety and that in your case, given the status of art, the best available variety happen to be modern varieties selected under a F1 hybrid breeding programme? Then of course, you will mention the caveat of F1 hybrids related to their genetic make up. But recent progress / Starmaya and superiority of these varieties is worth it.

 R : we modified the text according to the suggestion of the reviewer

*"Today, 90% of the world's Arabica plantations (more than seven million hectares) are planted with line varieties derived from a narrow genetic basis" *You might cite my paper: Montagnon, C., Mahyoub, A., Solano, W., & Sheibani, F. (2021). Unveiling a unique genetic diversity of cultivated Coffea arabica L. in its main domestication center: Yemen. Genetic Resources and Crop Evolution, 68(6), 2411-2422 ^3^ .

 R : we modified the text according to the suggestion of the reviewer and modifed the bibliography

*"In the majority of Arabica producing countries, there are no controlled or certified seed production bodies, putting farmers at risk that the seeds for new trees are not of consistent quality, genetic purity and variety standards"* You might cite my paper: Pruvot-Woehl, S., Krishnan, S., Solano, W., Schilling, T., Toniutti, L., Bertrand, B., & Montagnon, C. (2020). Authentication of Coffea arabica varieties through DNA fingerprinting and its significance for the coffee sector. *Journal of AOAC International*, *103*(2), 325-334 ^4^ .

 R : we modified the text according to the suggestion of the reviewer and modifed the bibliography

*"In Arabica, only the best individual of the best F1 hybrid progenies (...)"* : individual(S)

*"A Starmaya plant coming out of the nursery and ready to be planted costs about 1.2 times the cost of a plant produced by the producer with pure line seeds"*. I think you mean that it is cheap compared to clonal plants. This should be precise.

 R : we modified the text according to the suggestion of the reviewer

*"Voluntary sustainability standards (VSS) such as Fair Trade, which are becoming a sine qua non for access to specialty coffee markets, (...)"*: "Sine qua non" is a little bit strong. "are often associated with" sounds more correct.

 R : we modified the text according to the suggestion of the reviewer

*"the shift from diversified shade coffee to simplified shade or unshaded coffee (* *Goodall et al. 2015* *;* *Jha et al. 2014* *)"*.: I think there are more recent publications such as Harvey, C. A., Pritts, A. A., Zwetsloot, M. J., Jansen, K., Pulleman, M. M., Armbrecht, I., ... & Valencia, V. (2021). Transformation of coffee-growing landscapes across Latin America. A review. *Agronomy for sustainable development*, *41*(5), 1-19 ^5^ . R : we modified the text according to the suggestion of the reviewer and modified the bibliography

  " *La Cumplida also provides other farms with planting material and access to an innovative processing facility."* The point here is "High quality" planting material. 

 R : we modified the text according to the suggestion of the reviewer

*"(...) smallholders are currently discouraged by the low productivity of their variety (the Java variety) and low prices." *Be cautious on the Java variety which is not a poor variety in other context. Farming practices and neglecting the coffee plots is the main issue here.

 R : we modified the text according to the suggestion of the reviewer

*Figure 1a.* You might explicit in the text the reason why the NPV is zero for scenario 1 in the first two years. It is one of the main characteristic of this scenario.

 + in the capture you mention "F1 local variety" for scenario 2.

 R : we modified the text according to the suggestion of the reviewer

*Table 4*. Is the title correct?

 R : Yes

 (Discussion)

*"Especially for smallholder farmers, high quality premiums coupled with capacity building and access to i) highly productive varieties; ii) renovation of plantations; and iii) adapted credit with favourable (...)*." Be consistent with your qualification of varieties 'Highly productive"? Or "highly performing"? Or "Shade adapted"? Or other (resilient)? I think "Highly productive" is not giving justice to the message of the paper.

 R : we modified the text according to the suggestion of the reviewer

*"Differences in environmental conditions, climate, local practices etc. requires adapting some elements of the model. For instance, the shade level provided by associate trees has to be adapted to local conditions (generally between 30% and 40%) and take into account local preferences in timber and/or fruit trees. " *See my general comment of Ag practices which are not well documented and for which applied research is needed.

 R : we modified the text according to the suggestion of the reviewer

*"Pre-existing relationships with local stakeholders was detrimental in the success of the model in Nicaragua." *Detrimental?

 R : we modified the text according to the suggestion of the reviewer

*"a final roaster and a key intermediary organisation (ECOM),"* In fact, Ecom is the trader. Indeed, trader is at the interface between farmers and roasters.

 R : we modified the text according to the suggestion of the reviewer
